# Capturing the dynamics of adolescent emotion regulation in anxiety-inducing situations through development and evaluation of a state emotion regulation questionnaire for adolescents

**DOI:** 10.1038/s41598-025-17051-9

**Published:** 2025-09-01

**Authors:** Leonore Horváth, Nadine Vietmeier, Matthias Ziegler, Julian Schmitz, Brunna Tuschen-Caffier, Julia Asbrand

**Affiliations:** 1 Department of Psychology, Humboldt University Berlin, Berlin, Germany; 2 Department of Psychology, University of Leipzig, Leipzig, Germany; 3 Leipzig Research Centre for Early Childhood Development, Leipzig, Germany; 4 Department of Psychology, University of Freiburg, Freiburg, Germany; 5 German Centre for Mental Health, Site Halle-Jena-Magdeburg, Germany; 6 Department of Clinical Psychology of Childhood and Adolescence, Institute of Psychology, Friedrich Schiller University Jena, Semmelweisstr. 12, 07743 Jena, Germany

**Keywords:** Psychopathology, Child, Youth, Assessment, Risk factors, Therapeutics, Preclinical research

## Abstract

The development of functional emotion regulation (ER) is crucial for mental health in childhood and adolescence—especially in today’s context of multiple crises, which have led to rising anxiety even in the general population. Although the importance of ER is widely acknowledged, existing assessments have yet to adequately measure *state* ER, particularly in anxiety-inducing situations. We aimed to develop and evaluate the psychometric properties of the State Emotion Regulation Questionnaire (ERQ State) for adolescents, with a future focus on clinical populations. An online experiment was conducted with 105 German adolescents (*M*_age_ = 13.5 years, *SD*_age_ = 2.4), using four types of anxiety-inducing vignettes (social anxiety, general anxiety, specific phobia, separation anxiety) to examine potential correlations with trait anxiety. Confirmatory factor analyses were performed to refine the ERQ State-short item pool, revealing strong support for its theoretical structure in scenarios related to social anxiety (Vignette 1). However, model fit was less satisfactory for the remaining forms of anxiety (separation anxiety, specific phobia, generalized anxiety; Vignette 2). While further refinement and validation are needed, the ERQ State-short appears to be a promising tool for assessing ER in anxious adolescents, particularly in contexts involving social anxiety.

## Introduction

Emotion regulation (ER) is crucial for mental health^1^ and develops primarily during childhood and adolescence^2–4^. It is commonly conceptualized as a dynamic process involving the modulation of emotional responses to varying stimuli and contexts^5^. Given ER’s central role in both normative development and the risk of psychopathology^1^, there is a critical need for a psychometrically sound, emotion-specific questionnaire capable of measuring state ER in a context-dependent manner^6^.

Accurate and reliable measurement of ER is essential for advancing our understanding of child and youth development^7^. Adolescence represents a critical period of change in ER driven by evolving neurobiological and social contexts. Research indicates that peer interactions and pubertal development significantly influence emotional experiences^8^, while the use of adaptive ER strategies varies with age^9^—complicating overly simplistic models. Psychometrically robust ER assessments are therefore vital for studying youth mental health, identifying disorder-specific ER patterns for early detection and prevention, and evaluating the effectiveness of intervention^7^.

Effective ER is associated with improved social functioning, academic achievement, and psychological resilience, whereas ER deficits are linked to various psychopathological conditions—particularly anxiety disorders^10,11^. Maladaptive ER patterns may contribute to the persistence and worsening of anxiety symptoms^10^. Research has found that impaired ER is correlated with greater anxiety severity in youth (e.g.^12^) and can predict the onset of anxiety disorder^13^. In light of increasing anxiety levels amid ongoing global crises^14^, further investigation into ER as key mechanism^15,16^ in anxiety-inducing situations is critically needed.

Despite their widespread use in research and clinical settings, most ER assessments lack robust psychometric properties^7^. Moreover, they often require children to respond at a meta level (i.e., *how do you generally regulate negative emotions?*) overlooking both emotion specificity and situational context^17^. Additionally, issues such as projection and memory bias (e.g.^18^) raise concerns about the validity of trait-based ER assessment. Focusing on specific ER strategies within real or simulated situations may help address these limitations. While trait ER reflects individual differences across time and contexts, *state* ER captures transient, dynamic, and context-sensitive aspects of ER^19,20^.

However, existing evidence does not sufficiently support the psychometric quality of scores derived from situational ER questionnaires—when this methodological component has been examined at all (cf.^17^). To date, only a limited number of studies have investigated situation-specific ER in adolescence (open answers^21^; no psychometric report^22,23^). Addressing this gap is essential for developing effective interventions that promote adaptive regulation strategies and prevent maladaptive emotional responses^11^.

### The current study

The primary aim of this study was to develop and evaluate a state ER questionnaire specifically designed for adolescents in anxiety-inducing situations. To this end, we employed an experimental online methodology using case vignettes that simulated such situations (pre-registered at https://osf.io/8sahf). We pursued three main research objectives: First, we aimed to determine whether a confirmatory factor analysis (CFA) would support item loadings on superordinate factors corresponding to key ER strategies, including reappraisal, avoidance, rumination, acceptance, and distraction^24,25^. Second, we evaluated whether scores derived from the questionnaire would demonstrate at least acceptable internal consistency at the scale level, with Cronbach’s alpha coefficients exceeding 0.7^26^. Third, we assessed the questionnaire’s retest reliability by examining the moderate correlations of state ER responses across multiple situations—reflecting the short-term stability of ER strategies within individuals^27^. Additional exploratory analyses, which deviated from the preregistration, are reported in Supplementary Appendix A.

## Methods

### Participants

Data were collected as part of the project "Gefühle im Gleichgewicht: Neue methodische Ansätze im Kindes- und Jugendalter (Feelings in Balance: New methodological approaches in childhood and adolescence)”, conducted during the COVID-19 pandemic between January to December 2021. The study received ethics approval from the Institutional Review Board of the Department of Psychology at Humboldt-Universität zu Berlin (study number: 2020-65). Participants, along with their parents or legal guardians, received written information and provided informed consent prior to participation. The study was conducted in accordance with the principles outlined in the Declaration of Helsinki.

A structural equation model was computed for CFA (psychometric testing ERQ State). Sample size recommendations vary, ranging from simple rules of thumb (e.g., five times the number of estimated parameters) to more complex simulations (e.g.^28^). Given the pilot nature of the study, simulations were not conducted, and the commonly recommended sample size of *n* = 250 was adopted (e.g.^1,28^). Recruitment was carried out through schools, mailing lists, websites, student and parent committees, and social media platforms. As detailed in the pre-registration, data collection was limited by available time and personnel resources and was therefore concluded in December 2021. A total of *N* = 107 adolescents completed all questionnaires. Two participants were excluded due to consistently selecting the same response during the second administration of the ERQ State items. The final sample analyzed comprised *N* = 105 adolescents. To capture developmental differences across adolescence (cf.^7^), participants were recruited across a broad age range of 10 to 17 years. Exclusion criteria included incomplete informed consent and systematic response biases (e.g., consistently selecting the same response option). As a token of appreciation, participants were offered the chance to enter a raffle to win one of five 50€ gift cards.

### Design and procedure

**Fig. 1:**
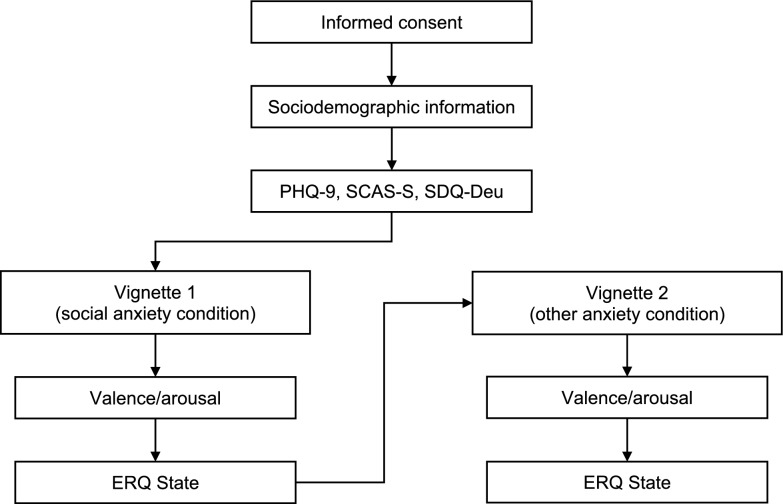
Experimental procedure. All steps, including questionnaire completion and the two experimental blocks (each involving a single vignette and subsequent ratings), were completed within one continuous session lasting approximately 60 min. PHQ-9 = Patient Health Questionnaire–9^29,30^. SCAS-S = Spence Children’s Anxiety Scale–Short^31^. SDQ-Deu = Strengths and Difficulties Questionnaire, German version^32^. ERQ State = State Emotion Regulation Questionnair.

The study employed a within-subjects design using the Humboldt-Universität zu Berlin version of LimeSurvey, an open-source survey platform (https://www.limesurvey.org/de). The primary within-subject factor was the type of vignette presented (see Fig. 1). Participants and their guardians received detailed information about the study, including confidentiality assurances and the right to withdraw. A contact address was provided for participant inquiries. Informed consent was obtained before participants provided sociodemographic data and completed various questionnaires assessing depressive symptoms, anxiety symptoms, and general psychopathology.

The entire procedure was conducted in a single experimental session lasting approximately one hour. The main experimental phase consisted of two blocks, each targeting different anxiety conditions through vignettes inspired by Carthy et al.^21^. In the first block, focused on social anxiety, participants were randomly assigned one vignette from a pool of five. They rated the vignette’s valence and arousal levels for the vignette before completing all 30 ERQ State items. The second block involved presenting a second vignette, randomly assigned to one of three anxiety conditions: separation anxiety, specific phobias, or generalized anxiety—with five or six vignettes for each condition.

### Materials and state measures

#### Vignettes

Building on the study by Carthy et al.^21^, we employed two sets of vignettes depicting potentially anxiety-inducing scenarios (see Supplementary Appendix B). Vignette 1 included situations related to social anxiety (e.g., "You are sitting in a group. The others introduce themselves briefly. You should introduce yourself in a moment."). Vignette 2 comprised scenarios with separation anxiety (e.g., "Your mother was supposed to come back from work, but she is late."), specific phobias (e.g., "A large dog is coming toward you on the street. It is heading straight for you."), or generalized anxiety (e.g., "On the way home from school, your stomach feels strange."). For each vignette, one scenario was randomly selected from a pool of five to six possibilities with equal probability. The vignettes were presented both auditorily and in written form.

#### ERQ State items

We based the development of the ERQ State on a synthesis of prior empirical investigations and theoretical frameworks. Initial exploration of state ER strategies in children aged 9 to 13 years, using Reappraisal and Suppression scales adapted from Egloff et al.^33^, revealed insufficient psychometric properties^17^. Following two pilot studies with university students and drawing on established theoretical models, e.g.^34^, established trait questionnaires^35,36^, and additional research findings, e.g.^37,38^, we developed an initial ER questionnaire. This questionnaire assessed five strategies—acceptance, avoidance, distraction, reappraisal, and rumination—with six items each (see Supplementary Appendix C for the full item list). This expanded item pool formed the basis for the ERQ State-Short, which includes three items per strategy, totaling 15 items. Participants indicated their level of agreement with each item on a 5-point scale (1 = *strongly disagree*; 2 = *disagree*; 3 = *neutral*; 4 = *agree*; 5 = *strongly agree*).

#### Ratings of arousal and valence

Arousal and valence ratings were assessed using visual analogue scales accompanied by visual stimuli, following the procedure of Carthy et al.^21^. Participants were asked to rate their current arousal level ("How tense do you feel at this moment?") on a scale from 1 (*very relaxed*) to 7 (*very tense*). Simultaneously, they rated their emotional valence ("How do you feel at this moment?") using the same 7-point scale from 1 (*very good*) to 7 (*very bad*).

### Additional measures: Traits

*SDQ-Deu.* The SDQ^32^ assesses behavioral symptoms across five scales: Emotional Symptoms, Conduct Problems, Hyperactivity/Inattention, Peer Problems, and Prosocial Behavior, each comprising five items. Participants respond on a 3-point scale (0 = *not true*; 1 = *somewhat true*; 2 = *certainly true*), with five reverse-coded items. Scale scores range from 0 to 10, where higher scores indicate greater difficulties, except for the Prosocial Behavior scale, where higher scores reflect strengths. The German SDQ (SDQ-Deu) has demonstrated good validity, showing strong correlations with the German Child Behavior Checklist^39^. In our sample, internal consistencies for the total difficulties score were robust (α = 0.81), while individual scales ranged from α = 0.53 (Conduct Problems scale) to α = 0.79 (Emotional Symptoms scale). Previous research has reported a wide range of internal consistency for SDQ subscales^40^, possibly due to symptom heterogeneity^41^.

#### SCAS-S

Anxiety symptoms were assessed using the German short version of the SCAS-S^31^, a concise alternative to the original SCAS^42^. It targets separation anxiety, social anxiety, panic, specific anxieties, and generalized anxiety. Participants rate the frequency of their anxiety symptoms on a 4-point scale (0 = *never*; 1 = *sometimes*; 2 = *often*; 3 = *always*), with higher scores indicating increased anxiety symptoms. The SCAS-S has demonstrated convergent and discriminant validity across multiple translations^43,44^, and shows good to excellent internal consistency for the total score (α = 0.88; ω = 0.93). In our sample, the German self-report SCAS-S exhibited excellent internal consistency for the total score (α = 0.90). Internal consistency for the subscales ranged from α = 0.47 (Separation Anxiety scale) to α = 0.88 (Panic Disorder scale).

#### PHQ-9

Depressive symptoms were assessed using the depression module of the PHQ-9^29,30^. Respondents rate each of nine items based on their experiences over the past 2 weeks using a 4-point scale (0 = *not at all*; 1 = *several days*; 2 = *more than half the days*; 3 = *nearly every day*). Total score ranges from 0 to 27, with higher scores indicating greater severity of depressive symptoms. Validation studies have demonstrated good internal consistency (α = 0.86; α = 0.89) and excellent test–retest reliability^29^. In a German sample, the PHQ-9 showed good internal consistency (α = 0.88^45^). Its validity has been supported by numerous studies, e.g.^29,45,46^, including those involving adolescents^47^. In our sample, the PHQ-9 demonstrated good internal consistency (α = 0.89).

### Data analysis

All analyses were performed using R Statistical Software (v4.2.1) and RStudio (v2022.12.0.353), primarily utilizing the packages psych (v2.2.9^48^), lavaan (v0.6.15^49^) and MBESS (v4.9.2^50^). Unless stated otherwise, a significance level of 0.05 was applied. For the second and third research objectives, analyses were conducted using the ERQ State-short, consisting of the final 15 items, to streamline computations and improve efficiency without compromising analytical rigor.

#### Research objective 1: Factorial structure

To reduce the extended ERQ State item pool into the concise ERQ State-short, consisting of three items per strategy, we conducted CFAs using lavaan (v0.6.15^49^). Measurement models were initially tested separately for each ER strategy to facilitate item reduction. The initial model for Vignette 1 included six ERQ State items per strategy along with their superordinate ER factors. When model fit was unsatisfactory, modification indices guided the identification of correlated residuals, and content-valid modifications were applied. Three items per strategy were selected based on criteria from Ziegler^51^, including factor loadings, residual correlations, item difficulty, and theoretical fit. Priority was given to items with high loadings, while also considering wording, distribution, and item similarity. Subsequently, three-item models were tested, and item patterns re-evaluated. A five-factor joint model was specified, allowing correlations among factors, and comparing fit indices and loadings. Multiple three-item models were explored as needed, replacing items with cross-loading. The final ERQ State-short version was chosen based on the best model fit after comparisons and modifications. Item selection was based on Vignette 1 data, and the resulting model was then tested against Vignette 2 data. For Vignette 2, anxiety conditions were collapsed into a single group due to limited observations per condition. Given Mardia’s test indicated nonnormality of the data, lavaan’s WLSMV was used. To address potential biases, the MLR estimator was also employed for comparison^52^. Model fit was assessed using the scaled χ^2^ statistic, where significant values indicate poor fit. Alternative fit indices included the (scaled) Comparative Fit Index (CFI), the (scaled) Root Mean Square Error of Approximation (RMSEA, for MLR only), and the Standardized Root Mean Square Residual (SRMR)—the Mplus-like SRMR for the MLR—with thresholds for good fit set at ≥ 0.95, ≤ 0.06, and ≤ 0.08, and ≥ 0.90, ≤ 0.08, and ≤ 0.08 for acceptable fit, respectively^53–55^. For the WLSMV, the Weighted Root Mean Square Residual (WRMR) was also examined with a cutoff of < 1.0 but interpreted cautiously due to the small and nonnormally distributed sample. A detailed description of the item reduction procedure and its results is provided in Supplementary Appendix D. Reported results refer exclusively to the final ERQ State-short.

#### Research objective 2: Internal consistencies

To evaluate the internal consistencies of the short scales, we used the MBESS package (v4.9.2^50^). In addition to Cronbach’s alpha^26^, we reported McDonald’s omega^56^, which is generally considered a more accurate estimate of reliability^57^. Specifically, we used the categorical version of omega (categorical omega; ω_cat_), as proposed by Green and Yang^58^, given its suitability for ordinal data^59^. Thresholds for acceptable reliability _cat_were set at ≥ 0.70 for both α and ω_cat_. Confidence intervals for the reliability estimates were calculated using bias-corrected and accelerated (BC*a*) bootstrapping, following recommendations by Kelley and Pornprasertmanit^60^, with 1,000 bootstrap iterations.

#### Research objective 3: Test–retest reliability

The test–retest reliability of the ERQ State-short scales was assessed across Vignettes 1 and 2, which were presented consecutively within the same experimental session, with all participants completing both the initial and follow-up measurements. Reliability was calculated using Spearman’s rank-order correlations. Bootstrap confidence intervals (BC*a* bootstrapping, 1,000 iterations) were computed using the confintr package (v1.0.0^61^). Holm adjustments were applied to correct for multiple testing.

## Results

### Participant characteristics

The final sample consisted of *N* = 105 adolescents aged 10 to 17 years (*M* = 13.5 years, *SD* = 2.4). Participants’ mothers had a mean age of 44.3 years (*SD* = 5.4), and fathers 45.8 years (*SD* = 8.3). Sociodemographic characteristics of the sample are summarized in Table 1. Due to participant withdrawal or exclusion, the final sample was no longer evenly distributed across the anxiety conditions in Vignette 2: *n* = 28 received a separation anxiety vignette (*M*_age_ = 13.5, *SD*_age_ = 2.5); *n* = 40 received a specific phobia vignette (*M*_age_ = 13.5, *SD*_age_ = 2.3); and *n* = 37 received a generalized anxiety vignette (*M*_age_ = 13.5, *SD*_age_ = 2.5). Participants’ age did not differ significantly between anxiety conditions for Vignette 2, *F*(2, 102) = 0.00, *p* = 0.999.

**Table 1:** Sociodemographic characteristics.

Variable	*n*	%
Gender		
Female	55	52.4
Male	45	42.9
Nonbinary	5	4.8
Living with		
Adoptive parents	1	1.0
Both parents	83	79.0
Mother (single parent)	10	9.5
Mother and partner	3	2.9
Father and partner	2	1.9
Other	6	5.7
Type of school		
Primary school	28	26.7
Comprehensive school (Gesamtschule) ^a^	7	6.7
Academic secondary school (Gymnasium) ^a^	66	62.9
Intermediate secondary school (Realschule)^a^	1	1.0
Other	3	2.9
Nationality		
German	97	92.4
Other	8	7.6
Country of birth^b^		
Germany	97	92.4
Other	7	6.7
(Primary) Language spoken at home		
German	99	94.3
Other	6	5.7
Community size		
1,000 to 5,000 residents	3	2.9
5,001 to 20,000 residents	10	9.5
20,001 to 100,000 residents	20	19.0
100,001 to 1,000,000 residents	14	13.3
More than 1,000,000 residents	58	55.2

*N* = 105. *M*_age_ = 13.5 years (*SD*_age_ = 2.4 years).

^a^Gesamtschule, Gymnasium, and Realschule are types of high schools in Germany.

^b^Response from one participant is missing.

### Manipulation check: Valence and arousal ratings

Participants reported mean valence levels of 3.2 (*SD* = 1.5) for Vignette 1 and 3.7 (*SD* = 1.9) for Vignette 2. A two-tailed paired *t*-test indicates that valence levels differed significantly between the two vignettes, *t*(104) =  − 2.46, *p* = 0.015, reflecting a small effect size, *d* =  − 0.24^62^. Further analysis of valence ratings within Vignette 2 revealed significant difference across the separation anxiety, specific phobias, and generalized anxiety conditions, *F*(2, 102) = 6.39, *p* = 0.002.

Post hoc pairwise *t*-tests using Benjamini–Hochberg adjustment indicated a significant difference between the specific phobias condition (*M* = 2.9, *SD* = 1.9) and both the generalized anxiety condition (*M* = 4.4, *SD* = 1.9), *p* = 0.002, and the separation anxiety condition (*M* = 4, *SD* = 1.8), *p* = 0.030. No significant difference was found between the separation anxiety and the generalized anxiety condition, *p* = 0.415. Mean arousal levels were identical for Vignette 1 and Vignette 2 (*M* = 3.7, *SD* = 1.7 and 1.9 resp.), with no significant difference detected in a two-tailed paired *t*-test, *t*(104) =  − 0.27, *p* = 0.790.

### Research objective 1: Factorial structure

**Fig. 2:**
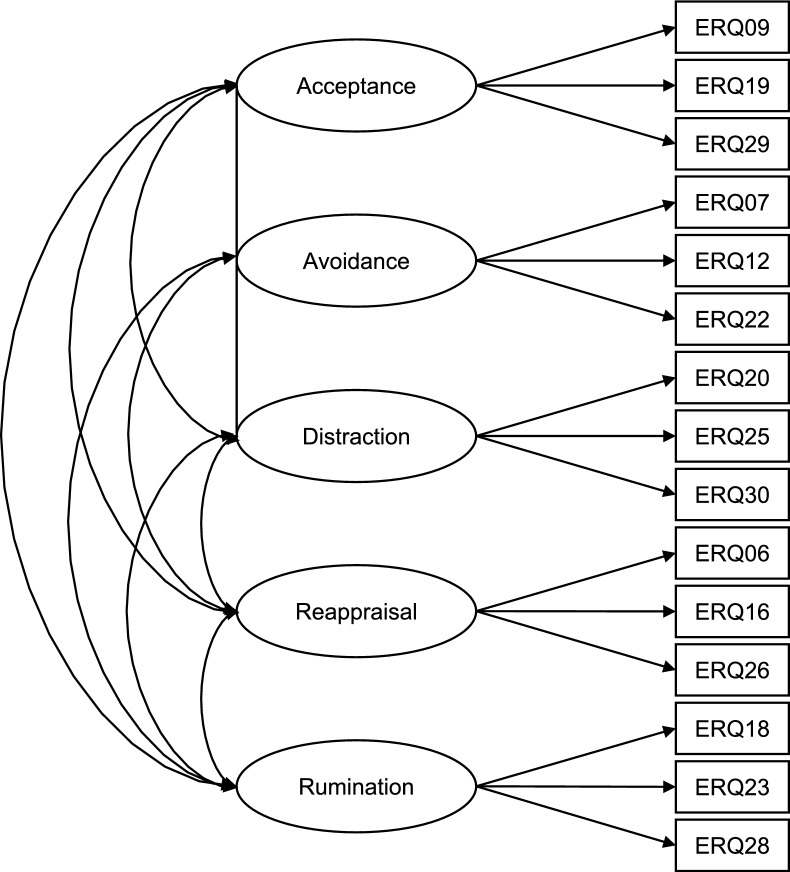
Schematic of the factorial structure of the final ERQ State-short. Since this figure is for visualization, estimates for factor loadings, factor correlations, and residuals are not shown. See Table 2 for factor loadings. ERQ numbers refer to the item numbers on the 30-item State Emotion Regulation Questionnaire.

Our first objective was to confirm the factorial structure of the final ERQ State-short following item reduction (see Supplementary Appendix D), using a CFA. Each ERQ State-Short item was specified to load onto its corresponding factor: acceptance, avoidance, distraction, reappraisal, or rumination. A schematic representation of the finalized model is presented in Fig. 2, while Table 2 displays the selected items along with their respective factor loadings. The complete initial item pool is provided in Supplementary Appendix C.

**Table 2:** Standardized factor loadings for the final state emotion regulation questionnaire, short form for each experimental condition.

ERQ State-short item	Vignette 1		Vignette 2	
	WLSMV	MLR	WLSMV	MLR
Strategy: acceptance				
ERQ09 I accept how I am feeling right now	0.83***	0.77***	0.91***	0.84***
ERQ19 I accept that I cannot change this feeling right now	0.47***	0.43**	0.46***	0.44**
ERQ29 It is okay to feel the way I do	0.79***	0.75***	0.89***	0.84***
Strategy: avoidance				
ERQ07 I wish I could have avoided the situation	0.74***	0.69***	0.73***	0.69***
ERQ12 I find the situation hard to bear	0.95***	0.90***	0.92***	0.84***
ERQ22 I would have liked to withdraw	0.88***	0.83***	0.86***	0.78***
Strategy: distraction				
ERQ20 I think of pleasant things that have nothing to do with the situation	0.72***	0.69***	0.91***	0.87***
ERQ25 I think about something other than how I feel	0.62***	0.56***	0.46***	0.41**
ERQ30 I think of something I enjoy doing	0.71***	0.67***	0.79***	0.71***
Strategy: reappraisal				
ERQ06 I try to make the best of the situation	0.71***	0.57**	0.41***	0.31
ERQ16 I tell myself that it is not really that bad	0.41***	0.51*	0.44***	0.57*
ERQ26 I tell myself that my value as a person does not depend on this	0.48***	0.48**	0.63***	0.41*
Strategy: rumination				
ERQ18 I feel like I have no control over my thoughts	0.88***	0.82***	0.87***	0.75***
ERQ23 I think, “Why do I always react this way?”	0.84***	0.79***	0.82***	0.78***
ERQ28 After the situation, I keep thinking about what happened	0.69***	0.65***	0.48***	0.46***

Translation of items from the original German for publication was conducted using deepl.com, then checked by the last author.* N* = 105. ERQ State-short = State Emotion Regulation Questionnaire; Vignette 1 = social anxiety condition; Vignette 2 = other anxiety condition; WLSMV = weighted least squares means and variance adjusted estimator method; MLR = robust maximum likelihood estimator.

*p < 0.05. **p < 0.01. ***p < 0.001.

For Vignette 1, the ERQ State-short yielded a significant χ^2^; however, the remaining fit indices indicated acceptable or near-acceptable model fit using the weighted least squares means and variance adjusted estimator (WLSMV; RMSEA excluded): χ^2^(80, *N* = 105) = 152.59, *p* < 0.001; CFI = 0.946; RMSEA = 0.093, 90% confidence interval (CI) [0.071, 0.116]; SRMR = 0.086; WRMR = 0.848. In contrast, the model demonstrated good fit under the robust MLR: χ^2^ (80, *N* = 105) = 98.88, *p* = 0.075; CFI = 0.960; RMSEA = 0.047, 90% CI [0.000, 0.075]; SRMR = 0.079. Across both estimation methods, all items loaded significantly onto their respective superordinate factors, with standardized loadings ranging from 0.41 to 0.95 (WLSMV) and from 0.43 to 0.90 (MLR) (see Table 1). These findings support the factorial validity of the ERQ State-short and affirm the first research objective. For Vignette 2, model fit was weaker than for Vignette 1 across both WLSMV and MLR, though some indices indicated acceptable fit under WLSMV. Specifically, SRMR exceeded the recommended threshold (RMSEA excluded): χ^2^(80, *N* = 105) = 181.49, *p* < 0.001; CFI = 0.926; RMSEA = 0.110, 90% CI [0.089, 0.132]; SRMR = 0.098; WRMR = 0.947. Under MLR, overall model fit did not meet acceptable criteria: χ^2^(80, *N* = 105) = 138.01, *p* < 0.001; CFI = 0.867; RMSEA = 0.083, 90% CI [0.059, 0.106]; SRMR = 0.090. With WLSMV, all items loaded significantly onto their intended superordinate factors, with standardized loadings ranging from 0.41 to 0.92. Using MLR, all items except ERQ06 demonstrated significant loadings, ranging from 0.41 to 0.87. Taken together, these findings offer weaker evidence for the factorial validity of the ERQ State-short in the context of Vignette 2, thereby providing only limited support for the first research objective.

### Research objective 2:Iinternal consistencies

To address our second research objective—evaluating the internal consistency of the ERQ State-short—we aimed for at least acceptable reliability for each subscale, defined as α ≥ 0.70 and ω₍cat₎ ≥ 0.70. Across both Vignette 1 and Vignette 2, the Acceptance, Avoidance, Distraction, and Rumination scales demonstrated acceptable or near-acceptable internal consistencies, as assessed by Cronbach’s alpha and ω₍cat₎ (see Table 3). In contrast, the Reappraisal scale failed to meet reliability thresholds under either condition, indicating insufficient internal consistency for that subscale.

**Table 3:** Internal consistencies for the short state emotion regulation questionnaire scales in each experimental condition.

Scale	Vignette 1				Vignette 2			
	α	95% CI_BC*a*_ [LL, UL]	ω_cat_	95% CI_BC*a*_ [LL, UL]	α	95% CI_BC*a*_ [LL, UL]	ω_cat_	95% CI_BC*a*_ [LL, UL]
Acceptance	0.68	[0.52, 0.78]	0.71	[0.55, 0.79]	0.75	[0.62, 0.84]	0.80	[0.69, 0.86]
Avoidance	0.85	[0.78, 0.90]	0.86	[0.79, 0.90]	0.82	[0.74, 0.87]	0.84	[0.77, 0.89]
Distraction	0.67	[0.49, 0.78]	0.69	[0.51, 0.79]	0.70	[0.54, 0.81]	0.74	[0.61, 0.82]
Reappraisal	0.53	[0.32, 0.68]	0.55	[0.33, 0.67]	0.40	[0.09, 0.57]	0.46	[0.11, 0.57]
Rumination	0.79	[0.67, 0.85]	0.81	[0.71, 0.86]	0.70	[0.56, 0.81]	0.74	[0.61, 0.83]

*N* = 105. Vignette 1 = social anxiety condition; Vignette 2 = other anxiety condition; ω_cat_ = categorical omega; CI_BC*a*_ = bias-corrected and accelerated bootstrap confidence interval (1,000 iterations); *LL* = lower limit; *UL* = upper limit.

### Research objective 3:Ttest–retest reliability

At least moderate correlations between the ERQ State-short scales across Vignettes 1 and 2 were expected (*r*_s_ > 0.30). The Acceptance, Distraction, and Rumination scales demonstrated moderate correlations (see Table 4), whereas the Avoidance and Reappraisal scales showed only small correlations between conditions. Therefore, our third research objective was achieved for the Acceptance, Distraction, and Rumination scales, but not for the Avoidance and Reappraisal scales.

**Table 4:** Spearman’s Rank-order correlations between the short state emotion regulation questionnaire scales across experimental conditions.

Scale	*r*_s_	95% CI_BC*a*_ [*LL*, *UL*]
Acceptance	0.39**	[0.20, 0.56]
Avoidance	0.24	[0.03, 0.43]
Distraction	0.45***	[0.25, 0.60]
Reappraisal	0.19	[− 0.01, 0.39]
Rumination	0.39**	[0.21, 0.56]

*N* = 105. Holm adjustments were applied to *p* values to correct for multiple testing. CI_BC*a*_ = bias-corrected and accelerated bootstrap confidence interval (1000 iterations); *LL* = lower limit; *UL* = upper limit.

**p < 0.01. ***p < 0.001.

## Discussion

In this study, our primary objective was to develop a concise questionnaire for assessing state ER strategies in anxiety-inducing situations among adolescents, focusing on acceptance, avoidance, distraction, reappraisal, and rumination as key ER strategies. By enabling the assessment of ER at the state level, we shifted from a general classification of strategies as adaptive or maladaptive toward a more dynamic perspective of ER, consistent with the evolving understanding of this complex process, e.g.^63,64^.

Using a CFA approach, we selected three items per ER strategy to create the 15-item ERQ State-short. Psychometric evaluation indicated a satisfactory factorial structure for Vignette 1 (social anxiety condition), but less optimal fit for Vignette 2 (other anxiety condition), which may be attributable to the heterogeneity of the second vignette. We deliberately chose one second vignette encompassing different anxiety scenarios to broaden the scope while keeping the assessment brief. The ERQ State-short’s poorer fit for Vignette 2 suggests potential challenges with item applicability across varied anxiety conditions. This finding underscores the need for caution when interpreting the questionnaire’s use across a broad range of anxiety scenarios. Furthermore, exploratory measurement invariance analyses revealed differential performance across vignettes (see Supplementary Appendix A.2), further advising against direct score comparisons. Although we adapted well-established vignettes from Carthy et al.^21^, which have demonstrated validity in capturing key aspects of anxiety disorders, some vignettes may be less robust given the broad age range in our sample. For example, prior research shows separation anxiety and specific phobias are more prevalent in early childhood, whereas social anxiety and generalized anxiety predominate in adolescence^65^. While our focus on social anxiety for Vignette 1 was thus appropriate, separation anxiety and specific phobia may be less relevant for this adolescent sample. To enhance the robustness of our findings, future research could develop new vignettes targeting anxiety problems more pertinent to directly compare on socially anxious and generalized anxious scenarios using the ERQ State-short. Additionally, more research employing ecological momentary assessment is necessary to extend state ER research beyond standardized vignettes to capture regulation in individual, real-world situations (e.g.^3^). The ERQ State-short could contribute to such studies and be further evaluated in these novel contexts.

Internal consistencies were generally acceptable, though the Reappraisal scale exhibited some shortcomings. Beyond measurement issues, the lower internal consistency of the Reappraisal scale may reflect the interaction between the strategy’s contextual variability and individual differences among adolescents^66^. For instance, adolescent girls might engage in cognitive reappraisal more frequently than boys, although the influence of age and gender remains unexplored^67^. Investigating the scale across different populations could clarify whether cultural or contextual factors (e.g., gender^68^; age^67^) affect internal consistency. In addition, future research could enhance the scale by refining item clarity and ensuring comprehensive coverage of all facets of the construct.

Scales test–retest correlations varied: Acceptance, Distraction, and Rumination demonstrated moderate stability, whereas Avoidance and Reappraisal showed lower stability. This suggests that dynamic ER strategies fluctuate based on situational and individual factors (e.g., developmental influences), as previously observed in trait ER strategies^24,69^. These findings underscore the complexity of dynamic ER in adolescents and point to important directions for future research. However, the limitations observed in the Reappraisal scale and the variability in strategy stability have significant implications for the overall validity of the questionnaire. In particular, inconsistencies in the Reappraisal and Distraction scales indicate a need for further refinement to improve the questionnaire’s robustness and to ensure it accurately reflects the diverse ER strategies adolescents employ. Future research should investigate the underlying causes of these inconsistencies and determine whether they arise from genuine individual differences or measurement issues.

Associations among ER strategies were observed (see Supplementary Appendix A), with theoretical explanations provided. However, the presence of high correlations raises concerns about potential overlap or methodological influences. This overlap may stem from conceptual issues, where strategies are less distinct than initially assumed, as well as methodological challenges, such as shared variance arising to measurement limitations. It is therefore critical to examine whether these strategies should be regarded as separate constructs or as interrelated components within a broader ER framework. Notably, the Avoidance and Reappraisal scales demonstrated insufficient test–retest reliability, possibly due to variations in anxiety conditions and issues specific to the Reappraisal scale. Furthermore, the potential lack of measurement invariance for the Avoidance scale suggests that its stability may have been affected by differing in anxiety conditions (see Supplementary Appendix A.2).

Our findings extend trait models on ER, e.g.^70^, and align with previous studies emphasizing the need of a comprehensive conceptual framework that incorporates flexible ER strategies^71–73^. Particular attention should be given to the role of distraction, as highlighted by our results and the inconclusive empirical evidence in prior research (see Supplementary Appendix D.2.3). The ambiguous role of distraction within ER frameworks may depend on factors such as the use of concurrent ER strategies or specific sample characteristics^74,75^, underscoring the need for further investigation. Future research should explore the validity of the ERQ State-short’s across a wider range of emotional contexts and assess its applicability in clinical samples. Additionally, refining the items within the Avoidance and Reappraisal scales could enhance their stability and relevance across anxiety conditions. Longitudinal studies may also help determine whether the observed issues with test–retest reliability stem from the inherent nature of the ER strategies or from limitations of the measurement itself.

It is important to emphasize that the ERQ State-short is not intended to replace existing trait measures of ER, e.g.^35,36,76,77^. Rather, it complements these instruments by providing valuable insights into state-level ER processes relevant to psychopathology, e.g.^34,78^. Despite its brevity, the ERQ State-short captures a broad range of commonly used ER strategies among adolescents^22,23^, thereby minimizing potential declines in response quality associated with longer assessments^79^. This feature may also enhance its utility in clinical settings, such as during exposure sessions. To explore the relationship between trait and state ER, and to further assess discriminant validity as an additional psychometric quality criterion, the ERQ State-short can be used as a time-efficient supplement to the ERQ trait measure.

In research, the ERQ State-short can contribute to a deeper understanding of the relationship between situation-specific strategy usage and psychopathology. Exploring the conditions under which strategies serve as protective or risk factors—considering aspects such as the specific emotions involved and the flexibility or rigidity of strategy use across situations—could yield valuable insights. Given the current scarcity of validated questionnaires in ecological momentary assessment studies involving adolescents^22,23^, the ERQ State-short has potential as a standardized tool for use in natural settings, facilitating better comparability of ER strategies across studies. Examining state ER across different anxiety disorders and comorbid conditions may uncover transdiagnostic patterns of dysregulation, providing insights into common mechanisms underlying various anxiety-related psychopathologies^80^. For example, research by Mathews et al.^81^ has identified specific associations between ER components and symptoms of social anxiety disorder and generalized anxiety disorder in adolescents. While social anxiety symptoms were linked to emotion understanding, acceptance, evaluation, and reactivity, generalized anxiety symptoms were uniquely related to emotion modification. Investigating distinct patterns of state-based ER could improve the tailoring of therapeutic approaches to specific strategies in various contexts^81^.

In treatment, understanding state ER in anxiety disorders could inform personalized treatment approaches. Interventions that target real-time emotion dysregulation may be more effective in reducing anxiety symptoms than those focused solely on trait-level interventions^82,83^. The ERQ States-short could eventually support therapy by identifying consistent patterns of strategy use across different contexts and emotions, including various therapeutic exercises. This information may help clinicians target and disrupt habits of inflexible strategy use, fostering greater ER flexibility. Simultaneously, assessing strategies over multiple therapy sessions could enable clinicians to monitor changes in strategy flexibility over time. Ultimately, the questionnaire has the potential to provide a more nuanced measurement of ER strategies by accounting for contextual variations as well as emotion- and psychopathology-specific mechanisms.

This study has some limitations that warrant consideration. First, the final sample size did not reach the originally intended target, posing methodological challenges, particularly for complex analyses such as CFAs and structural equation models (SEMs). Consequently, discriminant validity analyses between the ERQ State-short scales and measures of psychopathology within a CFA framework were not feasible. Although prior research suggests that SEM models can be evaluated with relatively small samples (*N* = 100 to 150^84^), smaller samples increase the risk of convergence failure, inaccurate parameter estimates, and unreliable model fit statistics^85^. While this exploratory study deemed the sample size acceptable, the reduction from the planned 250 to 105 participants may limit the robustness and generalizability of findings, especially regarding the ERQ’s factor structure. We therefore recommend replication with larger samples to confirm the dimensionality of the ERQ. Larger sample sizes are essential for more reliable analyses and to minimize potential issues with model fit and parameter estimation^86^. Second, participants were drawn exclusively from a nonclinical population, primarily attending a German *Gymnasium*-type school and residing in Berlin. This recruitment was influenced by logistical considerations, such as partnerships with local institutions and ease of access. Despite efforts to include participants from diverse regions and school types, the sample may not fully represent the broader demographic diversity of Germany. Adolescents from different geographical and socio-cultural contexts (e.g., urban vs. rural areas^87^) may interpret emotional scenarios differently due to cultural, social, and environmental influences. Thus, generalizability to clinical populations or more diverse educational and life backgrounds is limited, underscoring the need for further research in varied populations. Finally, due to the ongoing pandemic during data collection, online assessments were used, introducing inherent limitations such as reduced control of participants’ testing environment. To mitigate this, participants were encouraged to share their thoughts at the survey’s conclusion. Future studies might benefit from incorporating multimethod approaches—such as behavioral and physiological measures—and conducting assessments in controlled settings to gain a more comprehensive understanding of emotion regulation strategies.

In summary, the ERQ State-short holds promise for both research and clinical applications by illuminating the dynamic nature of adolescent ER, particularly in social anxiety contexts. It enables a more nuanced examination of temporal changes in strategy use and their adaptation to diverse situations. However, before widespread adoption, comprehensive investigations are necessary to address potential issues—such as those observed with the Reappraisal and Distraction scales—including low internal consistencies, cross-loadings, and inadmissible solutions. A more detailed examination of the ERQ State-short across different anxiety conditions, specific age groups, and genders could further clarify its suitability for capturing ER strategies unique to each condition. As the understanding of adolescent ER continues to develop, this instrument offers a foundation for further exploration, offering insights into how adolescents navigate their emotional experiences, especially in anxiety-inducing scenarios.

## Supplementary Information

Supplementary Information.

## Data Availability

The data that support the findings of this study are available from first authors, but restrictions apply to the availability of these data, which were used under license for the current study, and so are not publicly available. Data are, however, available from the authors upon reasonable request and with permission of the last author.

## References

[1] Compas, B. E. et al. Coping, emotion regulation, and psychopathology in childhood and adolescence: A meta-analysis and narrative review. *Psychol. Bull.* **143**, 939–991 (2017). 28616996 10.1037/bul0000110 PMC7310319

[2] Morris, A. S., Silk, J. S., Steinberg, L., Myers, S. S. & Robinson, L. R. The role of the family context in the development of emotion regulation. *Soc. Dev.* **16**, 361–388 (2007). 19756175 10.1111/j.1467-9507.2007.00389.x PMC2743505

[3] Morris, A. S., Criss, M. M., Silk, J. S. & Houltberg, B. J. The impact of parenting on emotion regulation during childhood and adolescence. *Child Dev. Perspect.* **11**, 233–238 (2017).

[4] Thompson, R. A. Emotion regulation: A theme in search of definition. *Monogr. Soc. Res. Child Dev.* **59**, 25–52 (1994). 7984164

[5] Cole, P. M., Michel, M. K. & Teti, L. O. The development of emotion regulation and dysregulation: A clinical perspective. *Monogr. Soc. Res. Child Dev.* **59**, 73–100 (1994). 7984169

[6] Zeman, J., Cassano, M., Perry-Parrish, C. & Stegall, S. Emotion regulation in children and adolescents. *J. Dev. Behav. Pediatr.* **27**, 155 (2006). 16682883 10.1097/00004703-200604000-00014

[7] Mazefsky, C. A. et al. Evidence base update for questionnaires of emotion regulation and reactivity for children and adolescents. *J. Clin. Child Adolesc. Psychol.* **50**, 683–707 (2021). 34436940 10.1080/15374416.2021.1955372 PMC10205090

[8] Guyer, A. E., Caouette, J. D., Lee, C. C. & Ruiz, S. K. Will they like me? Adolescents’ emotional responses to peer evaluation. *Int. J. Behav. Dev.* **38**, 155–163 (2014). 25076803 10.1177/0165025413515627 PMC4112521

[9] Zimmermann, P. & Iwanski, A. Emotion regulation from early adolescence to emerging adulthood and middle adulthood: Age differences, gender differences, and emotion-specific developmental variations. *Int. J. Behav. Dev.* **38**, 182–194 (2014).

[10] Suveg, C. & Zeman, J. Emotion regulation in children with anxiety disorders. *J. Clin. Child Adolesc. Psychol.* **33**, 750–759 (2004). 15498742 10.1207/s15374424jccp3304_10

[11] Muris, P. & Ollendick, T. H. The role of temperament in the etiology of child psychopathology. *Clin. Child Fam. Psychol. Rev.* **8**, 271–289 (2005). 16362256 10.1007/s10567-005-8809-y

[12] Golombek, K., Lidle, L., Tuschen-Caffier, B., Schmitz, J. & Vierrath, V. The role of emotion regulation in socially anxious children and adolescents: A systematic review. *Eur. Child. Adolesc. Psychiatry* **29**, 1479–1501 (2020). 31201527 10.1007/s00787-019-01359-9

[13] Beesdo, K., Knappe, S. & Pine, D. S. Anxiety and anxiety disorders in children and adolescents: Developmental issues and implications for DSM-V. *Psychiatr. Clin.* **32**, 483–524 (2009). 10.1016/j.psc.2009.06.002 PMC3018839 19716988

[14] Ravens-Sieberer, U. et al. Quality of life and mental health in children and adolescents during the first year of the COVID-19 pandemic: Results of a two-wave nationwide population-based study. *Eur. Child Adolesc. Psychiatry* **32**, 575–588 (2023). 34636964 10.1007/s00787-021-01889-1 PMC8506100

[15] Daniel, S. K., Abdel-Baki, R. & Hall, G. B. The protective effect of emotion regulation on child and adolescent wellbeing. *J. Child Fam. Stud.* **29**, 2010–2027 (2020).

[16] Marchi, J., Johansson, N., Sarkadi, A. & Warner, G. The impact of the COVID-19 pandemic and societal infection control measures on children and adolescents’ mental health: A scoping review. *Front. Psychiatry* **1**, 711791. 10.3389/fpsyt.2021.711791 (2021). 10.3389/fpsyt.2021.711791 PMC8451953 34552516

[17] Asbrand, J., Heinrichs, N., Schmidtendorf, S., Nitschke, K. & Tuschen-Caffier, B. Experience versus report: Where are changes seen after exposure-based cognitive-behavioral therapy? A randomized controlled group treatment of childhood social anxiety disorder. *Child Psychiatry Hum. Dev.* **51**, 427–441 (2020). 31960175 10.1007/s10578-019-00954-w PMC7235054

[18] Chang, V. T., Overall, N. C., Madden, H. & Low, R. S. T. Expressive suppression tendencies, projection bias in memory of negative emotions, and well-being. *Emotion* **18**, 925–941 (2018). 29389201 10.1037/emo0000405

[19] Silvers, J. A. et al. Age-related differences in emotional reactivity, regulation, and rejection sensitivity in adolescence. *Emotion* **12**, 1235–1247 (2012). 22642356 10.1037/a0028297 PMC4131311

[20] Sheppes, G., Suri, G. & Gross, J. J. Emotion regulation and psychopathology. *Annu. Rev. Clin. Psychol.* **11**, 379–405 (2015). 25581242 10.1146/annurev-clinpsy-032814-112739

[21] Carthy, T., Horesh, N., Apter, A., Edge, M. D. & Gross, J. J. Emotional reactivity and cognitive regulation in anxious children. *Behav. Res. Ther.* **48**, 384–393 (2010). 20089246 10.1016/j.brat.2009.12.013

[22] Lennarz, H. K., Hollenstein, T., Lichtwarck-Aschoff, A., Kuntsche, E. & Granic, I. Emotion regulation in action: Use, selection, and success of emotion regulation in adolescents’ daily lives. *Int. J. Behav. Dev.* **43**, 1–11 (2018). 30613118 10.1177/0165025418755540 PMC6305959

[23] Tan, P. Z. et al. Emotional reactivity and regulation in anxious and nonanxious youth: A cell-phone ecological momentary assessment study. *J. Child Psychol. Psychiatry* **53**, 197–206 (2011). 22176136 10.1111/j.1469-7610.2011.02469.x PMC3258378

[24] Gross, J. J. & John, O. P. Individual differences in two emotion regulation processes: Implications for affect, relationships, and well-being. *J. Pers. Soc. Psychol.* **85**, 348–362 (2003). 12916575 10.1037/0022-3514.85.2.348

[25] McRae, K. et al. The development of emotion regulation: An fMRI study of cognitive reappraisal in children, adolescents and young adults. *Soc. Cogn. Affect. Neurosci.* **7**, 11–22 (2012). 22228751 10.1093/scan/nsr093 PMC3252634

[26] Cronbach, L. J. Coefficient alpha and the internal structure of tests. *Psychometrika* **16**, 297–334 (1951).

[27] Kraemer, H. C., Stice, E., Kazdin, A., Offord, D. & Kupfer, D. How do risk factors work together? Mediators, moderators, and independent, overlapping, and proxy risk factors. *AJP* **158**, 848–856 (2001). 10.1176/appi.ajp.158.6.848 11384888

[28] Kline, R. B. *Principles and Practice of Structural Equation Modeling* (The Guilford Press, 2016).

[29] Kroenke, K., Spitzer, R. L. & Williams, J. B. W. The PHQ-9: Validity of a brief depression severity measure. *J. Gen. Intern. Med.* **16**, 606–613 (2001). 11556941 10.1046/j.1525-1497.2001.016009606.x PMC1495268

[30] Löwe, B., Kroenke, K., Herzog, W. & Gräfe, K. Measuring depression outcome with a brief self-report instrument: Sensitivity to change of the Patient Health Questionnaire (PHQ-9). *J. Affect. Disord.* **81**, 61–66 (2004). 15183601 10.1016/S0165-0327(03)00198-8

[31] Ahlen, J., Vigerland, S. & Ghaderi, A. Development of the spence children’s anxiety scale-short version (SCAS-S). *J. Psychopathol. Behav. Assess.* **40**, 288–304 (2018). 29937623 10.1007/s10862-017-9637-3 PMC5978831

[32] Goodman, R. The strengths and difficulties questionnaire: A research note. *J. Child Psychol. Psychiatry* **38**, 581–586 (1997). 9255702 10.1111/j.1469-7610.1997.tb01545.x

[33] Egloff, B., Schmukle, S. C., Burns, L. R. & Schwerdtfeger, A. Spontaneous emotion regulation during evaluated speaking tasks: Associations with negative affect, anxiety expression, memory, and physiological responding. *Emotion* **6**, 356–366 (2006). 16938078 10.1037/1528-3542.6.3.356

[34] Aldao, A. & Nolen-Hoeksema, S. Specificity of cognitive emotion regulation strategies: A transdiagnostic examination. *Behav. Res. Ther.* **48**, 974–983 (2010). 20591413 10.1016/j.brat.2010.06.002

[35] Grob, A. & Smolenski, C. *Fragebogen zur Erhebung der Emotionsregulation bei Kindern und Jugendlichen: FEEL-KJ (Emotion Regulation Questionnaire for Children and Adolescents)* (Hogrefe, 2005).

[36] Gullone, E. & Taffe, J. The emotion regulation questionnaire for children and adolescents (ERQ–CA): A psychometric evaluation. *Psychol. Assess.* **24**, 409–417 (2012). 22023559 10.1037/a0025777

[37] Asbrand, J., Svaldi, J., Krämer, M., Breuninger, C. & Tuschen-Caffier, B. Familial accumulation of social anxiety symptoms and maladaptive emotion regulation. *PLoS ONE* **11**, e0153153 (2016). 27055278 10.1371/journal.pone.0153153 PMC4824435

[38] Keil, V., Asbrand, J., Tuschen-Caffier, B. & Schmitz, J. Children with social anxiety and other anxiety disorders show similar deficits in habitual emotional regulation: Evidence for a transdiagnostic phenomenon. *Eur. Child Adolesc. Psychiatry* **26**, 749–757 (2017). 28078476 10.1007/s00787-017-0942-x

[39] Klasen, H. et al. Comparing the German versions of the strengths and difficulties questionnaire (SDQ-Deu) and the child behavior checklist. *Eur. Child Adolesc. Psychiatry* **9**, 271–276 (2000). 11202102 10.1007/s007870070030

[40] Stone, L. L., Otten, R., Engels, R. C. M. E., Vermulst, A. A. & Janssens, J. M. A. M. Psychometric properties of the parent and teacher versions of the strengths and difficulties questionnaire for 4- to 12-year-olds: A review. *Clin. Child Fam. Psychol. Rev.* **13**, 254–274 (2010). 20589428 10.1007/s10567-010-0071-2 PMC2919684

[41] McCrae, R. R., Kurtz, J. E., Yamagata, S. & Terracciano, A. Internal consistency, retest reliability, and their implications for personality scale validity. *Pers. Soc. Psychol. Rev.* **15**, 28–50 (2011). 20435807 10.1177/1088868310366253 PMC2927808

[42] Spence, S. H. A measure of anxiety symptoms among children. *Behav. Res. Ther.* **36**, 545–566 (1998). 9648330 10.1016/s0005-7967(98)00034-5

[43] Essau, C. A., Sasagawa, S., Anastassiou-Hadjicharalambous, X., Guzmán, B. O. & Ollendick, T. H. Psychometric properties of the Spence Child Anxiety Scale with adolescents from five European countries. *J. Anxiety Disord.* **25**, 19–27 (2011). 20685072 10.1016/j.janxdis.2010.07.001

[44] Essau, C. A., Muris, P. & Ederer, E. M. Reliability and validity of the spence children’s anxiety scale and the screen for child anxiety related emotional disorders in German children. *J. Behav. Ther. Exp. Psychiatry* **33**, 1–18 (2002). 12389796 10.1016/s0005-7916(02)00005-8

[45] Gräfe, K., Zipfel, S., Herzog, W. & Löwe, B. Screening psychischer Störungen mit dem Gesundheitsfragebogen für Patienten (PHQ-D). *Diagnostica* **50**, 171–181 (2004).

[46] Löwe, B. et al. Comparative validity of three screening questionnaires for DSM-IV depressive disorders and physicians’ diagnoses. *J. Affect. Disord.* **78**, 131–140 (2004). 14706723 10.1016/s0165-0327(02)00237-9

[47] Richardson, L. P. et al. Evaluation of the patient health questionnaire-9 item for detecting major depression among adolescents. *Pediatrics* **126**, 1117–1123 (2010). 21041282 10.1542/peds.2010-0852 PMC3217785

[48] Revelle, W. *psych: Procedures for Personality and Psychological Research* (2022).

[49] Rosseel, Y. lavaan: An R package for structural equation modeling. *J. Stat. Softw.* **48**, 1–36 (2012).

[50] Kelley, K. *MBESS: The MBESS R Package* (2022).

[51] Ziegler, M. Comments on item selection procedures. *Eur. J. Psychol. Assess.* **30**, 1–2 (2014).

[52] Finney, S., DiStefano, C. & Kipp, J. P. Overview of estimation methods and preconditions for their application with structural equation modeling. In *Principles and Methods of Test Construction: Standards and Recent Advances* (Hogrefe, 2016).

[53] Gerosa, T. *Measurement Invariance with Ordered Categorical Variables. Applications in Longitudinal Survey Research* (2021).

[54] Hu, L. & Bentler, P. M. Cutoff criteria for fit indexes in covariance structure analysis: Conventional criteria versus new alternatives. *Struct. Equ. Modeling* **6**, 1–55 (1999).

[55] Hu, L. & Bentler, P. M. Fit indices in covariance structure modeling: Sensitivity to underparameterized model misspecification. *Psychol. Methods* **3**, 424–453 (1998).

[56] McDonald, R. P. *Test Theory—A Unified Treatment* (Psychology Press).

[57] McNeish, D. Thanks coefficient alpha, we’ll take it from here. *Psychol. Methods* **23**, 412–433 (2018). 28557467 10.1037/met0000144

[58] Green, S. B. & Yang, Y. Reliability of summed item scores using structural equation modeling: An alternative to coefficient alpha. *Psychometrika* **74**, 155–167 (2009).

[59] Viladrich, C., Angulo-Brunet, A. & Doval, E. A journey around alpha and omega to estimate internal consistency reliability. *Psicol-Spain* **33**, 755 (2017).

[60] Kelley, K. & Pornprasertmanit, S. Confidence intervals for population reliability coefficients: Evaluation of methods, recommendations, and software for composite measures. *Psychol. Methods* **21**, 69–92 (2016). 26962759 10.1037/a0040086

[61] Mayer, M. *confintr: Confidence Intervals* (2023).

[62] Cohen, J. *Statistical Power Analysis for the Behavioral Sciences* (Routledge, 1988).

[63] Aldao, A., Sheppes, G. & Gross, J. J. Emotion regulation flexibility. *Cogn. Ther. Res.* **39**, 263–278 (2015).

[64] Blanke, E. S. et al. Mix it to fix it: Emotion regulation variability in daily life. *Emotion* **20**, 473–485 (2020). 30714776 10.1037/emo0000566

[65] Steinsbekk, S., Ranum, B. & Wichstrøm, L. Prevalence and course of anxiety disorders and symptoms from preschool to adolescence: A 6-wave community study. *Child Psychol. Psychiatry* **63**, 527–534 (2022). 10.1111/jcpp.13487 34318492

[66] Lennarz, H. K., Hollenstein, T., Lichtwarck-Aschoff, A., Kuntsche, E. & Granic, I. Emotion regulation in action: Use, selection, and success of emotion regulation in adolescents’ daily lives. *Int. J. Behav. Dev.* **43**, 1–11 (2019). 30613118 10.1177/0165025418755540 PMC6305959

[67] Willner, C. J. et al. The development of cognitive reappraisal from early childhood through adolescence: A systematic review and methodological recommendations. *Front. Psychol.* **13**, 875964 (2022). 35814075 10.3389/fpsyg.2022.875964 PMC9258621

[68] Johnson, D. P. & Whisman, M. A. Gender differences in rumination: A meta-analysis. *Person. Individ. Differ.* **55**, 367–374 (2013). 10.1016/j.paid.2013.03.019 PMC3786159 24089583

[69] Aldao, A., Nolen-Hoeksema, S. & Schweizer, S. Emotion-regulation strategies across psychopathology: A meta-analytic review. *Clin. Psychol. Rev.* **30**, 217–237 (2010). 20015584 10.1016/j.cpr.2009.11.004

[70] Gross, J. J. The extended process model of emotion regulation: Elaborations, applications, and future directions. *Psychol. Inq.* **26**, 130–137 (2015).

[71] Katz, B. A., Lustig, N., Assis, Y. & Yovel, I. Measuring regulation in the here and now: The development and validation of the State Emotion Regulation Inventory (SERI). *Psychol. Assess.* **29**, 1235–1248 (2017). 27936820 10.1037/pas0000420

[72] Lee, D. J., Witte, T. K., Weathers, F. W. & Davis, M. T. Emotion regulation strategy use and posttraumatic stress disorder: Associations between multiple strategies and specific symptom clusters. *J. Psychopathol. Behav. Assess.* **37**, 533–544 (2015).

[73] Naragon-Gainey, K., McMahon, T. P. & Chacko, T. P. The structure of common emotion regulation strategies: A meta-analytic examination. *Psychol. Bull.* **143**, 384–427 (2017). 28301202 10.1037/bul0000093

[74] Lyubomirsky, S., Tucker, K. L., Caldwell, N. D. & Berg, K. Why ruminators are poor problem solvers: Clues from the phenomenology of dysphoric rumination. *J. Pers. Soc. Psychol.* **77**, 1041–1060 (1999). 10573879 10.1037//0022-3514.77.5.1041

[75] Wolgast, M. & Lundh, L.-G. Is distraction an adaptive or maladaptive strategy for emotion regulation? A person-oriented approach. *J. Psychopathol. Behav. Assess.* **39**, 117–127 (2017). 28286372 10.1007/s10862-016-9570-x PMC5323484

[76] Junghänel, M. et al. Validation of a new emotion regulation self-report questionnaire for children. *BMC Psychiatry* **22**, 820 (2022). 36550484 10.1186/s12888-022-04440-x PMC9773459

[77] Rådman, G., Claréus, B. & Daukantaitė, D. Adolescents’ emotion regulation strategies questionnaire–extended: Further development and associations with mental health problems in adolescence. *Assessment* **31**, 482–501 (2024). 37056041 10.1177/10731911231164619 PMC10822064

[78] Lougheed, J. P. & Hollenstein, T. A limited repertoire of emotion regulation strategies is associated with internalizing problems in adolescence. *Soc. Dev.* **21**, 704–721 (2012).

[79] Galesic, M. & Bosnjak, M. Effects of questionnaire length on participation and indicators of response quality in a web survey. *Public Opin. Q.* **73**, 349–360 (2009).

[80] Barlow, D. H., Raffa, S. D. & Cohen, E. M. Psychosocial treatments for panic disorders, phobias, and generalized anxiety disorder. In *A Guide to Treatment that Works* 301–336 (Oxford University Press, 2002).

[81] Mathews, B. L., Kerns, K. A. & Ciesla, J. A. Specificity of emotion regulation difficulties related to anxiety in early adolescence. *J. Adolesc.* **37**, 1089–1097 (2014). 25150890 10.1016/j.adolescence.2014.08.002

[82] Roy-Byrne, P. et al. Delivery of evidence-based treatment for multiple anxiety disorders in primary care: A randomized controlled trial. *JAMA* **303**, 1921–1928 (2010). 20483968 10.1001/jama.2010.608 PMC2928714

[83] Kendall, P. C. & Treadwell, K. R. H. The role of self-statements as a mediator in treatment for youth with anxiety disorders. *J. Consult. Clin. Psychol.* **75**, 380–389 (2007). 17563155 10.1037/0022-006X.75.3.380

[84] Kyriazos, T. A. Applied psychometrics: Sample size and sample power considerations in factor analysis (EFA, CFA) and SEM in general. *PSYCH* **09**, 2207–2230 (2018).

[85] Wang, J. & Wang, X. *Structural Equation Modeling: Applications Using Mplus* (Wiley, 2012).

[86] Kretzschmar, A. & Gignac, G. E. At what sample size do latent variable correlations stabilize? *J. Res. Pers.* **80**, 17–22 (2019).

[87] Vitale, V. & Bonaiuto, M. The role of nature in emotion regulation processes: An evidence-based rapid review. *J. Environ. Psychol.* **96**, 102325 (2024).

