# Supplementary material for: Capturing the dynamics of adolescent emotion regulation in anxiety-inducing situations through development and evaluation of a state emotion regulation questionnaire for adolescents
Source: Sci Rep. 2025 Sep 1;15:32231. doi: 10.1038/s41598-025-17051-9 (PMC12402453; doi:10.1038/s41598-025-17051-9)
Supplement: Supplementary file 1 — Supplementary Information. [file 41598_2025_17051_MOESM1_ESM.docx]

**Capturing the Dynamics of Adolescent Emotion Regulation in Anxiety-inducing Situations through Development and Evaluation of a State Emotion Regulation Questionnaire for Adolescents**

# **Appendix A - Explorative analyses**

## A.1 Discriminative validity

The following analyses on discriminant validity were not part of the pre-registration and are therefore presented here. To further investigate the psychometric properties of the ERQ State-Short, we hypothesized that the ERQ State-Short scales would be empirically distinguishable both (a) from one another and (b) from measures of psychopathology.

## A.1.1 Associations Among the ERQ State-Short Scales

Discriminant validity of the ERQ State-Short scales was assessed using confirmatory factor analysis (CFA) with the WLSMV estimator by examining factor correlations and comparing their confidence interval boundaries to the cut-off criteria proposed by Rönkkö and Cho¹. Table A1 presents the factor correlations and corresponding confidence intervals for each experimental condition.

**Table A1**

*Means, Standard Deviations, and Factor Correlations of the ERQ State-Short Scales Within Each Experimental Condition*

| Scale | *M* | *SD* | Acceptance | | Avoidance | Distraction | | Reappraisal | Rumination |
| --- | --- | --- | --- | --- | --- | --- | --- | --- | --- |
| Vignette 1 | | | | | | | | | |
| Acceptance | 10.6 | 2.7 | — | |  |  | |  |  |
| Avoidance | 8.3 | 3.6 | −.70^***^  [−.82, −.58] | | — |  | |  |  |
| Distraction | 8.3 | 2.9 | .23^*^  [.01, .46] | | .08  [−.15, .31] | — | |  |  |
| Reappraisal | 10.0 | 2.8 | .66^***^  [.48, .85] | −.54^***^  [−.74, −.34] | | | .45^***^  [.20, .70] | — |  |
| Rumination | 8.2 | 3.6 | −.72^***^  [−.84, −.59] | | .90^***^  [.82, .97] | .20  [.00, .41] | | −.52^***^  [−.75, −.30] | — |
| Vignette 2 | | | | | | | | | |
| Acceptance | 10.8 | 3.0 | — | |  |  | |  |  |
| Avoidance | 8.1 | 3.8 | −.71^***^  [−.82, −.60] | | — |  | |  |  |
| Distraction | 8.4 | 3.2 | .21^*^  [.03, .39] | | .17  [−.03, .37] | — | |  |  |
| Reappraisal | 9.3 | 3.0 | .77^***^  [.51, 1.03] | −.59^***^  [−.84, −.34] | | | .59^***^  [.37, .82] | — |  |
| Rumination | 7.3 | 3.4 | −.65^***^  [−.77, −.52] | | .84^***^  [.74, .94] | .40^***^  [.20, .60] | | −.40^**^  [−.67, −.13] | — |

*Note. N* = 105. Factor correlations were computed using WLSMV. The 95% confidence intervals are presented in brackets, with the first value indicating the lower limit (*LL*) and the second value indicating the upper limit (*UL*). Vignette 1 = social anxiety condition; Vignette 2 = other anxiety condition.

^*^*p* < .05. ^**^*p* < .01. ^***^*p* < .001.

For Vignette 1, the confidence interval upper limits (UL) for the correlations between acceptance and avoidance, acceptance and reappraisal, and acceptance and rumination indicated marginal discriminant validity issues (.80 ≤ UL < .90); for negative correlations, the UL refers to the absolute value of the lower limit. The UL for the correlation between avoidance and rumination suggested a moderate discriminant validity problem (.90 ≤ UL < 1). All other correlations showed no discriminant validity concerns (UL < .80). Thus, discriminant validity for the ERQ State-Short scales during Vignette 1 was only partially supported.

For Vignette 2, the confidence interval ULs for correlations between reappraisal and avoidance, reappraisal and distraction, and acceptance and avoidance indicated marginal discriminant validity problems (.80 ≤ UL < .90). The UL for avoidance and rumination suggested a moderate problem (.90 ≤ UL < 1), while the UL for acceptance and reappraisal indicated a severe discriminant validity issue (UL ≥ 1). No problems were observed with the remaining correlations (UL < .80). Therefore, discriminant validity could not be assumed for all ERQ State-Short scales during Vignette 2.

### A.1.2 Associations with Measures of Psychopathology

Discriminant validity of the ERQ State-Short scales in relation to psychopathology measures was evaluated using Spearman’s rank-order correlations corrected for attenuation. Correlations below .80 were interpreted as indicating no discriminant validity concerns. For both Vignette 1 (see Table A2) and Vignette 2 (see Table A3), all correlations remained below this threshold, supporting the assumption of discriminant validity.

**Table A2**

*Corrected Spearman's Rank-Order Correlations Between the ERQ State-Short Scales and Measures of Psychopathology During Vignette 1 (Social Anxiety Condition)*

| Variable | Acceptance | Avoidance | Distraction | Reappraisal | Rumination |
| --- | --- | --- | --- | --- | --- |
| PHQ-9 | −.58  [−.77, −.33] | .59  [.39, .77] | −.27  [−.51, −.01] | −.48  [−.72, −.18] | .62  [.41, .78] |
| SCAS-S |  |  |  |  |  |
| Total score | −.48  [−.67, −.25] | .63  [.45, .78] | −.04  [−.29, .22] | −.27  [−.53, .03] | .65  [.43, .78] |
| Separation anxiety | −.23  [−.59, .13] | .26  [−.07, .55] | .09  [−.26, .45] | −.06  [−.45, .34] | .38  [.06, .69] |
| Social anxiety | −.69  [−.85, −.50] | .65  [.46, .80] | −.10  [−.37, .14] | −.46  [−.72, −.18] | .68  [.51, .84] |
| Panic disorder | −.48  [−.68, −.26] | .51  [.33, .69] | −.08  [−.33, .18] | −.35  [−.62, −.06] | .61  [.42, .77] |
| Specific phobias | −.08  [−.39, .25] | .58  [.31, .79] | .12  [−.21, .42] | .09  [−.27, .46] | .51  [.23, .77] |
| Generalized anxiety | −.47  [−.72, −.22] | .66  [.42, .86] | −.05  [−.33, .24] | −.23  [−.57, .11] | .63  [.36, .85] |
| SDQ-Deu |  |  |  |  |  |
| Total difficulties score | −.54  [−.74, −.28] | .67  [.49, .84] | −.17  [−.44, .11] | −.35  [−.63, −.06] | .72  [.52, .89] |
| Emotional symptoms | −.51  [−.72, −.26] | .70  [.50, .85] | −.08  [−.34, .18] | −.31  [−.59, −.02] | .72  [.51, .86] |
| Conduct problems | −.42  [−.72, −.13] | .24  [−.03, .50] | −.20  [−.55, .15] | −.36  [−.73, .01] | .48  [.19, .74] |
| Hyperactivity | −.22  [−.50, .06] | .40  [.15, .64] | −.07  [−.37, .24] | −.03  [−.37, .34] | .33  [.03, .58] |
| Peer problems | −.56  [−.82, −.27] | .62  [.39, .82] | −.06  [−.37, .27] | −.52  [−.80, −.17] | .68  [.43, .87] |
| Prosocial behaviour | .27  [−.03, .54] | −.07  [−.33, .18] | −.09  [−.37, .24] | .37  [.00, .66] | −.12  [−.39, .15] |

*Note. N* = 105. The 95% confidence intervals, presented in brackets, were calculated using bias-corrected and accelerated bootstrapping with 1,000 iterations. The first value represents the lower limit (*LL*) and the second value represents the upper limit (*UL*). All correlations and corresponding confidence intervals were corrected for attenuation based on Cronbach’s alpha. PHQ-9 = Patient Health Questionnaire-9; SCAS-S = Spence Children’s Anxiety Scale-Short; SDQ-Deu = Strengths and Difficulties Questionnaire.

**Table A3**

*Corrected Spearman's Rank-Order Correlations Between the ERQ State-Short Scales and Measures of Psychopathology During Vignette 2 (Other Anxiety Condition)*

| Variable | Acceptance | Avoidance | Distraction | Reappraisal | Rumination |
| --- | --- | --- | --- | --- | --- |
| PHQ-9 | −.40  [−.60, −.16] | .34  [.09, .54] | .03  [−.23, .26] | −.18  [−.51, .13] | .42  [.18, .65] |
| SCAS-S |  |  |  |  |  |
| Total score | −.24  [−.48, .00] | .33  [.12, .54] | .19  [−.04, .41] | .01  [−.32, .35] | .52  [.31, .76] |
| Separation anxiety | −.19  [−.50, .18] | .30  [−.01, .60] | .28  [−.07, .58] | .06  [−.35, .55] | .46  [.07, .74] |
| Social anxiety | −.33  [−.57, −.07] | .29  [.05, .53] | .05  [−.23, .35] | −.09  [−.44, .26] | .40  [.15, .65] |
| Panic disorder | −.24  [−.45, .00] | .24  [.02, .47] | .13  [−.10, .36] | −.07  [−.37, .30] | .41  [.14, .62] |
| Specific phobias | −.18  [−.45, .12] | .41  [.13, .68] | .27  [−.03, .53] | .10  [−.31, .54] | .60  [.26, .85] |
| Generalized anxiety | −.20  [−.48, .11] | .37  [.11, .63] | .17  [−.13, .44] | −.12  [−.54, .24] | .61  [.31, .85] |
| SDQ-Deu |  |  |  |  |  |
| Total difficulties score | −.37  [−.58, −.11] | .31  [.07, .53] | .04  [−.22, .28] | −.24  [−.59, .06] | .51  [.28, .72] |
| Emotional symptoms | −.30  [−.53, .07] | .39  [.13, .59] | .28  [.02, .52] | −.10  [−.46, .24] | .54  [.30, .76] |
| Conduct problems | −.23  [−.53, .09] | .03  [−.24, .34] | −.14  [−.47, .19] | −.22  [−.67, .19] | .37  [.09, .65] |
| Hyperactivity | −.26  [−.51, .04] | .27  [.01, .52] | .03  [−.25, .31] | −.43  [−.77, −.07] | .36  [.04, .59] |
| Peer problems | −.29  [−.59, .03] | .07  [−.23, .36] | −.07  [−.39, .25] | −.09  [−.49, .33] | .24  [−.07, .55] |
| Prosocial behaviour | −.11  [−.37, .16] | .28  [.01, .51] | .00  [−.27, .27] | .18  [−.23, .56] | .06  [−.20, .36] |

*Note. N* = 105. The 95% confidence intervals, presented in brackets, were calculated using bias-corrected and accelerated bootstrapping with 1,000 iterations. The first value represents the lower limit (*LL*) and the second value represents the upper limit (*UL*). All correlations and corresponding confidence intervals were corrected for attenuation based on Cronbach’s alpha. PHQ-9 = Patient Health Questionnaire-9; SCAS-S = Spence Children’s Anxiety Scale-Short; SDQ-Deu = Strengths and Difficulties Questionnaire.
A.2 Explorative Measurement Invariance Analyses

A.2.1 Data Analyses

Measurement invariance for the configural, loading, and intercept models was tested using the MLR estimator by stepwise introducing model restrictions ^2,3^. Specifically, equality constraints were applied to the same factor structure, factor loadings, and intercepts ^4,5^. If invariance holds across these conditions, it allows for comparisons of factor means and variances ^2,6^. More constrained models were compared to less constrained models using the chi-square difference test (Δχ²) and changes in CFI (∆CFI; ^6^). A non-significant Δχ² and ∆CFI ≤ −.01 suggest the presence of invariance ^6^.

For ordinal data, threshold and loading invariance are prerequisites for comparing scores across groups ^7^. Using the WLSMV estimator, configural, threshold, and loading invariance models were tested as recommended by Wu and Estabrook ^2^ and Svetina et al. ^5^. Model comparisons for WLSMV were again conducted using Δχ² ^4^, as changes in alternative fit indices are not recommended when using WLSMV ^3,4,7^. Results of both estimators were considered, as the analyses were exploratory.

A.2.2 Results

*WLSMV*

**Acceptance Scale.** The configural model for the Acceptance scale showed a good fit (see Table A4 for fit indices and test statistics from the measurement invariance analyses). Imposing threshold constraints did not result in a statistically significant difference from the configural model, and no significant difference was observed between the subsequent loading invariance model and the threshold invariance model. Thus, configural, threshold, and loading invariance were assumed for the Acceptance scale using WLSMV.

**Avoidance Scale.** The configural model for the Avoidance scale showed good fit. Further imposition of threshold constraints did not significantly worsen the fit. Testing for loading did not reject the hypothesis of invariant loadings and therefore, configural, threshold, and loading invariance were assumed for the Avoidance scale using WLSMV.

**Distraction Scale.** The configural model for the Distraction scale showed a good fit. Constraining thresholds to be equal over time resulted in a model with a significant χ^2^ value. However, the model fit was not significantly worse than the configural model according to the Δχ². Exploratory inspection of potential modifications revealed that no modification would significantly improve the model fit, so no threshold constraint was freed. Additional loading constraints did not result in a significant loss of fit. Thus, configural, threshold, and loading invariance were assumed for the Distraction scale using WLSMV.

**Rumination Scale.** The configural model for the Reappraisal scale showed a good fit. The imposition of threshold and loading constraints did not significantly deteriorate the fit according to the Δχ². Therefore, configural, threshold, and loading invariance were assumed for the Reappraisal scale using WLSMV.

**Rumination Scale.** The configural model for the Rumination scale showed a good fit, and imposing threshold invariance did not significantly worsen the model fit according to the Δχ². However, imposing loading constraints led to a significant difference between the threshold invariance model and the loading invariance model. Specifically, item ERQ28 (“Ich denke im Anschluss an die Situation ganz viel darüber nach, was passiert ist.”) loaded more strongly onto the rumination factor during Vignette 1 (social anxiety condition) than during Vignette 2 (other anxiety condition). Freeing the loading constraint for this item resulted in a model fit statistically indistinguishable from the threshold invariance model. Thus, configural, threshold, and partial loading invariance were assumed for the Rumination scale using WLSMV.

**Table A4**

*Measurement Invariance Analyses Between Both Experimental Conditions for the ERQ State- Short Using WLSMV*

| Invariance  model | χ2 | *df* | CFI | RMSEA  90% CI [*LL*, *UL*] | SRMR | WRMR | Δχ² | Δ*df* |
| --- | --- | --- | --- | --- | --- | --- | --- | --- |
|  |  |  |  | Acceptance |  |  |  |  |
| Configural | 4.58 | 5 | 1.000 | .000 [.000, .130] | .033 | 0.266 |  |  |
| Threshold | 12.75 | 11 | .997 | .039 [.000, .114] | .033 | 0.406 | 9.13 | 6 |
| Loading | 13.66 | 13 | .999 | .022 [.000, .101] | .035 | 0.431 | 1.20 | 2 |
|  |  |  |  | Avoidance |  |  |  |  |
| Configural | 8.64 | 5 | .995 | .084 [.000, .175] | .041 | 0.317 |  |  |
| Threshold | 11.94 | 11 | .999 | .029 [.000, .109] | .041 | 0.339 | 2.02 | 6 |
| Loading | 18.09 | 13 | .993 | .061 [.000, .123] | .045 | 0.444 | 5.23 | 2 |
|  |  |  |  | Distraction |  |  |  |  |
| Configural | 9.81 | 5 | .988 | .096 [.000, .185] | .042 | 0.367 |  |  |
| Threshold | 21.23^*^ | 11 | .974 | .095 [.028, .155] | .042 | 0.502 | 11.69 | 6 |
| Loading | 18.29 | 13 | .987 | .063 [.000, .124] | .042 | 0.511 | 0.45 | 2 |
|  |  |  |  | Reappraisal |  |  |  |  |
| Configural | 7.14 | 5 | .967 | .064 [.000, .161] | .049 | 0.363 |  |  |
| Threshold | 12.84 | 11 | .972 | .040 [.000, .115] | .049 | 0.403 | 4.17 | 6 |
| Loading | 14.14 | 13 | .983 | .029 [.000, .104] | .052 | 0.440 | 1.52 | 2 |
|  |  |  |  | Rumination |  |  |  |  |
| Configural | 6.57 | 5 | .996 | .055 [.000, .155] | .039 | 0.290 |  |  |
| Threshold | 11.24 | 11 | .999 | .014 [.000, .104] | .039 | 0.325 | 3.28 | 6 |
| Loading | 21.37 | 13 | .980 | .079 [.000, .136] | .044 | 0.483 | 8.09^*^ | 2 |
| Partial  loading | 11.10 | 12 | 1.000 | .000 [.000, .094] | .039 | 0.326 | 0.07 | 1 |

*Note. N* = 105. CFI = comparative fit index; RMSEA = root mean square error of approximation; CI = confidence interval; *LL* = lower limit; *UL* = upper limit; SRMR = standardized root mean square residual; WRMR = weighted root mean square residual; Δχ² = chi-square difference test; Δ*df* = difference in degrees of freedom between two nested models.

^*^*p* < .05. ^**^*p* < .01. ^***^*p* < .001.

*MLR*

**Acceptance Scale.** The configural model for the Acceptance scale produced a good fit (see Table A5 for fit indices and test statistics from the measurement invariance analyses), and further addition of loading and intercept constraints did not result in a significant deterioration of fit. Thus, configural, loading, and intercept invariance were assumed for the Acceptance scale using MLR.

**Avoidance Scale.** The configural model for the Avoidance scale produced an acceptable fit according to CFI and SRMR, but an insufficient fit according to RMSEA and χ². Exploratory inspection of modification indices suggested correlated residuals between ERQ12 (“Es fällt mir schwer die Situation auszuhalten.”) from Vignette 1 and ERQ22 (“Ich hätte mich gerne zurückgezogen.”) from Vignette 2. As these correlations could not be theoretically justified, and given the exploratory nature of the analysis, the model was retained without modifications. Imposing loading constraints led to a significant decline in fit according to Δχ² and ∆CFI. After freeing the loading constraint for ERQ22 (“Ich hätte mich gerne zurückgezogen.”), the partially loading invariant model did not show a statistically worse fit compared to the configural model. Further imposition of intercept constraints did not lead to a significant deterioration in fit. Thus, the model indicated partial loading invariance for ERQ22, as it loaded more strongly onto the avoidance factor during Vignette 1 compared to Vignette 2.

**Distraction Scale.** The configural model for the Distraction scale showed a good fit. Introducing loading and intercept restrictions did not result in a significant decline in model fit compared to the previous model. Therefore, configural, loading, and intercept invariance were assumed for the Distraction scale using MLR.

**Reappraisal Scale.** The configural model for the Reappraisal scale showed a good fit. The Δχ² supported the assumption of invariant loadings; however, ∆CFI did not. As a result, constraints for invariant loadings of ERQ26 (“Ich sage mir, dass mein Wert als Person davon nicht abhängt.”) across both conditions were freed. The resulting partially loading invariant model did not show a significantly worse fit compared to the configural model according to both Δχ² and ∆CFI. However, the subsequently tested invariance model exhibited a significant deterioration in fit compared to the partially loading invariant model. Freeing the intercept constraint for ERQ06 (“Ich versuche, das Beste aus der Situation zu machen.”) improved model fit. The resulting partially intercept model did not show a significantly worse fit compared to the partially loading invariant model. Therefore, partial loading and intercept invariance were established for the Reappraisal scale using MLR: Specifically, ERQ26 loaded more strongly onto the reappraisal factor during Vignette 1, and the intercept for ERQ06 was higher during Vignette 1 compared to Vignette 2.

**Rumination Scale.** The configural model for the Rumination scale showed a good fit. After adding loading constraints, the fit did not worsen significantly according to Δχ² and ∆CFI. However, further addition of intercept constraints resulted in a significant deterioration of fit. Freeing the intercept constraint for ERQ28 (“Ich denke im Anschluss an die Situation ganz viel darüber nach, was passiert ist.”) led to a partially intercept invariant model that showed an acceptable fit, though it still differed significantly from the loading invariant model. Additionally, freeing the intercept constraint for ERQ23 (“Ich denke, ‘Warum reagiere ich immer so?’”) resulted in a model that did not produce a worse fit than the loading invariance model. Further inspection showed that intercepts for ERQ23 and ERQ28 were higher for Vignette 1. Since two out of three items failed to demonstrate intercept invariance, only loading invariance was assumed for the Rumination scale using MLR.

**Table A5**

*Measurement Invariance Analyses Between Both Experimental Conditions for the ERQ State- Short Using MLR*

| Invariance model | χ2 | *df* | CFI | RMSEA 90% CI [*LL*, *UL*] | SRMR | Δχ² | Δ*df* | ΔCFI |
| --- | --- | --- | --- | --- | --- | --- | --- | --- |
|  |  |  |  | Acceptance |  |  |  |  |
| Configural | 3.75 | 5 | 1.000 | .000 [.000, .103] | .032 |  |  |  |
| Loading | 4.72 | 7 | 1.000 | .000 [.000, .082] | .037 | 0.88 | 2 | .000 |
| Intercept | 8.87 | 10 | 1.000 | .000 [.000, .090] | .048 | 4.36 | 3 | .000 |
|  |  |  |  | Avoidance |  |  |  |  |
| Configural | 12.99^*^ | 5 | .965 | .123 [.036, .213] | .037 |  |  |  |
| Loading | 22.25^**^ | 7 | .933 | .144 [.075, .219] | .058 | 9.43^**^ | 2 | –.032 |
| Partial loading | 14.10^*^ | 6 | .964 | .113 [.029, .196] | .045 | 1.26 | 1 | –.001 |
| Intercept | 19.47^*^ | 9 | .954 | .105 [.037, .171] | .055 | 5.57 | 3 | –.010 |
|  |  |  |  | Distraction |  |  |  |  |
| Configural | 5.73 | 5 | .993 | .037 [.000, .137] | .034 |  |  |  |
| Loading | 6.41 | 7 | 1.000 | .000 [.000, .107] | .036 | 0.72 | 2 | .007 |
| Intercept | 8.85 | 10 | 1.000 | .000 [.000, .092] | .042 | 2.42 | 3 | .000 |
|  |  |  |  | Reappraisal |  |  |  |  |
| Configural | 5.66 | 5 | .982 | .036 [.000, .151] | .040 |  |  |  |
| Loading | 9.57 | 7 | .930 | .059 [.000, .148] | .051 | 3.94 | 2 | –.052 |
| Partial  loading | 5.02 | 6 | 1.000 | .000 [.000, .115] | .040 | 0.05 | 1 | .018 |
| Intercept | 17.11^*^ | 9 | .778 | .093 [.010, .159] | .080 | 12.28^**^ | 3 | –.222 |
| Partial intercept | 6.42 | 8 | 1.000 | .000 [.000, .098] | .043 | 1.40 | 2 | .000 |
|  |  |  |  | Rumination |  |  |  |  |
| Configural | 4.76 | 5 | 1.000 | .000 [.000, .131] | .032 |  |  |  |
| Loading | 6.80 | 7 | 1.000 | .000 [.000, .117] | .047 | 2.04 | 2 | .000 |
| Intercept | 24.78^**^ | 10 | .894 | .119 [.060, .179] | .080 | 19.92^***^ | 3 | –.106 |
| Partial intercept | 14.29 | 9 | .962 | .075 [.000, .145] | .059 | 8.48^*^ | 2 | –.038 |
| Partial intercept (2) | 6.80 | 8 | 1.000 | .000 [.000, .102] | .047 | 0.02 | 1 | .000 |

*Note. N* = 105. CFI = comparative fit index; RMSEA = root mean square error of approximation; CI = confidence interval; *LL* = lower limit; *UL* = upper limit; SRMR = standardized root mean square residual; Δχ² = chi-square difference test; Δ*df* = difference in degrees of freedom between two nested models; ΔCFI = difference in CFI between two nested models.

## ^*^*p* < .05. ^**^*p* < .01. ^***^*p* < .001.

## A.3 Explorative Inspection of Other Joint Models.

Upon finalizing the selection of the ERQ State-Short items, alternative joint models incorporating these items were explored for Vignette 1. Four models were examined: a hierarchical model with a superordinate ER factor (Model B), variations of a two-factor adaptive-maladaptive model (Model C), a two-factor cognitive-behavioral model (Model D), and variations of a model based on Gross’ process model (Model E). Both WLSMV and MLR estimators were used to assess these models. A detailed description of the models and the strategies employed within them can be found in Appendix E. All alternative models (Models B to E) were compared to the initially tested five-factor model (Model A) using the chi-square difference test (Δ_χ²_). The results of this exploratory inspection are provided in greater detail in Appendix E.

# **Appendix B – Vignettes**

**Table B1**

*Anxiety vignettes*

|  | **German Original** | **English Translation** |
| --- | --- | --- |
| Social Anxiety | Du sitzt in einer Gruppe. Die anderen stellen sich kurz vor. Gleich sollst du dich gleich vorstellen. | You are sitting in a group. The others are briefly introducing themselves. Soon it will be your turn to introduce yourself. |
|  | Du siehst einige deiner Mitschülerinnen und Mitschüler. Du gehst zu ihnen rüber. Als du näherkommst, hörst du sie lachen. | You see some of your classmates. You walk over to them. As you get closer, you hear them laughing. |
|  | Heute ist Schulfest. Du trittst mit einer Hauptrolle in einem Theaterstück auf. Du hast es gründlich geübt, und nun schauen dich alle Leute erwartungsvoll an. | Today is the school party. You have a leading role in a play. You have practiced thoroughly, and now everyone is looking at you with anticipation. |
|  | Auf dem Nachhauseweg siehst du eine Gruppe älterer Kinder/Jugendlicher. Sie albern herum und kommen auf dich zu. | On your way home, you see a group of older children/teenagers. They are goofing around and walking towards you. |
|  | Dein Klassenlehrer kündigt für nächste Woche eine Klassenarbeit an. | Your teacher announces a class test for next week. |
| Separation Anxiety | Deine Mutter sollte von der Arbeit zurückkommen, aber sie ist zu spät. | Your mother was supposed to come back from work, but she is late. |
|  | Deine Eltern wollten abends um 22 Uhr von einer Feier zurückkommen. Es ist 23 Uhr und sie sind noch nicht zuhause. | Your parents were supposed to return from a party at 10 pm. It is now 11 pm, and they are still not home yet. |
|  | Deine Mutter geht zur Nachbarin. Nach einer Weile merkst du, dass sie noch immer nicht zurückgekommen ist. | Your mother goes to the neighbor's house. After a while, you realize that she still has not come back. |
|  | Du machst mit deinen Eltern einen Ausflug. Während einer Pause schaust du dir allein die Umgebung an. Plötzlich merkst du, dass du den Weg zu deinen Eltern nicht mehr weißt. | You are on an outing with your parents. During a break, you explore the area on your own. Suddenly, you realize you no longer know the way back to your parents. |
|  | Du bist mit deinen Eltern in einer fremden Stadt. Du schaust dir ein Schaufenster an, und als du dich umdrehst, siehst du deine Eltern nicht mehr. | You are in an unfamiliar city with your parents. You are looking at a shop window, and when you turn around, your parents are gone. |
| Specific Phobia | Auf der Straße kommt dir ein großer Hund entgegen. Er läuft genau auf dich zu. | A large dog is coming toward you on the street. It is heading straight for you. |
|  | Du machst mit deinen Eltern einen Ausflug. Ihr seht einen hohen Aussichtsturm. Deine Eltern wollen mit dir hinaufsteigen. | You are on an outing with your parents. You see a tall observation tower, and your parents want to climb it with you. |
|  | Du möchtest gerne Radfahren. Dein Fahrrad steht im dunklen Keller. | You want to ride your bike. It is in the dark basement. |
|  | Du spielst am Nachmittag in deinem Zimmer. Plötzlich fängt es an zu blitzen, und ein lauter Donner ist zu hören. | You are playing in your room in the afternoon. Suddenly, it starts to lighten and thunder loudly. |
|  | Du bist beim Arzt. Der soll dich einmal gründlich untersuchen. Er möchte dir etwas Blut abnehmen. | You are at the doctor’s office for a thorough check-up. They want to draw some blood. |
|  | Du räumst dein Zimmer auf. Hinter einer Kiste entdeckst du eine Spinne. | You are cleaning up your room. Behind a box, you find a spider. |
| Generalized Anxiety | In den Nachrichten wird darüber gesprochen, dass es einen Terroranschlag in einer Stadt in deiner Nähe gab. | The news reports a terrorist attack in a nearby city. |
|  | Du liegst abends im Bett und kannst vor lauter Gedanken im Kopf nicht schlafen. | You are lying in bed at night, and you cannot sleep because of all the thoughts running through your head. |
|  | Im Radio hörst du von einem Zugunglück in deiner Nachbarstadt. | You hear on the radio about a train accident in your neighboring town. |
|  | Du hast verschlafen. In zehn Minuten beginnt der Unterricht und du weißt, dass du mindestens 15min zu spät sein wirst. | You overslept. Class starts in ten minutes, and you know you will be at least 15 minutes late. |
|  | Auf dem Weg von der Schule nach Hause fühlst du dich komisch im Bauch. | On the way home from school, your stomach feels strange. |

# **Appendix C – Complete List of Items**

**Table C1**

*Complete list of emotion regulation items^^[[1]](#footnote-2)^^*

|  | **Item number** | **German original** | **English translation** |
| --- | --- | --- | --- |
| **Acceptance** | ERQ04 (R) | Ich kann mich nur schwer damit abfinden, wie es mir gerade geht. | I find it hard to come to terms with how I am feeling right now. |
|  | ERQ09 | Ich akzeptiere, wie es mir gerade geht. | I accept how I am feeling right now. |
|  | ERQ14 | Ich denke mir, dass es vorbei gehen wird. | I tell myself that it will pass. |
|  | ERQ19 | Ich akzeptiere, dass ich das Gefühl jetzt nicht ändern kann. | I accept that I cannot change this feeling right now. |
|  | ERQ24 | Ich denke, dass ich die Situation akzeptieren muss. | I think that I have to accept the situation. |
|  | ERQ29 | Es ist ok, wie ich mich fühle. | It is ok to feel the way I do. |
| **Avoidance** | ERQ02 | Ich würde am liebsten der Situation entfliehen. | I would most like to escape the situation. |
|  | ERQ07 | Ich hätte die Situation gerne vermieden. | I wish I could have avoided the situation. |
|  | ERQ12 | Es fällt mir schwer die Situation auszuhalten. | I find the situation hard to bear. |
|  | ERQ17 | Ich würde dafür sorgen, nicht in eine solche Situation zu kommen. | I would make sure not to get into such a situation. |
|  | ERQ22 | Ich hätte mich gerne zurückgezogen. | I would have liked to withdraw. |
|  | ERQ27 | Ich würde zukünftig solche Situationen vermeiden. | I would avoid such situations in the future. |
| **Distraction** | ERQ05 | Ich lenke mich in Gedanken von der Situation hier ab. | I distract myself mentally from the situation. |
|  | ERQ10 | Ich stelle mir vor, woanders zu sein. | I imagine being somewhere else. |
|  | ERQ15(R) | Ich bin völlig konzentriert auf die Situation hier. | I am completely focused on the current situation. |
|  | ERQ20 | Ich denke an angenehme Dinge, die nichts mit der Situation zu tun haben. | I think of pleasant things that have nothing to do with the situation. |
|  | ERQ25 | Ich denke an etwas anderes als daran, wie ich mich fühle. | I think about something other than how I feel. |
|  | ERQ30 | Ich denke an etwas, das ich gerne tue. | I think of something I enjoy doing. |
| **Reappraisal** | ERQ01 | Ich versuche, an der Situation etwas Gutes zu finden. | I try to find something positive in the situation. |
|  | ERQ06 | Ich versuche, das Beste aus der Situation zu machen. | I try to make the best of the situation. |
|  | ERQ11 | Ich versuche, die Situation als Herausforderung zu sehen. | I try to see the situation as a challenge. |
|  | ERQ16 | Ich sage mir, dass das alles doch gar nicht so schlimm ist. | I tell myself that it is not really that bad. |
|  | ERQ21 | Ich sage mir, dass ich es beim nächsten Mal besser machen kann. | I tell myself that I can do better next time. |
|  | ERQ26 | Ich sage mir, dass mein Wert als Person davon nicht abhängt. | I tell myself that my value as a person does not depend on this. |
| **Rumination** | ERQ03 | Ich überlege immer wieder, warum ich mich gerade so fühle, wie ich mich fühle. | I keep thinking about why I feel how I feel right now. |
|  | ERQ08 | Ich denke immer wieder darüber nach, was jetzt passieren könnte. | I keep thinking about what might happen next. |
|  | ERQ13(R) | Ich denke nicht besonders über meine Gefühle nach. | I do not think much about my feelings. |
|  | ERQ18 | Ich habe das Gefühl, keine Kontrolle über meine Gedanken zu haben. | I feel like I have no control over my thoughts. |
|  | ERQ23 | Ich denke, „Warum reagiere ich immer so?“ | I think, ”Why do I always react this way?” |
|  | ERQ28 | Ich denke im Anschluss an die Situation ganz viel darüber nach, was passiert ist. | After the situation, I keep thinking about what happened. |

# **Appendix D – Item Reduction Procedure**

D.1 Data Analysis

For item reduction, measurement models were initially tested separately for each ER strategy. Each strategy included all six ERQ State items from the extended item pool, along with their corresponding superordinate ER factors. Measurement models were computed only for Vignette 1 at this stage. In cases where model fit was not acceptable, modification indices were examined, allowing for correlated residuals between items within the same ER strategy. Modifications were implemented only if they were meaningfully aligned with the content of the items.

Three items per ER strategy were selected when an acceptable model fit was achieved. As suggested by Ziegler ^8^, several criteria were applied in this selection process. Items were chosen based on their loadings, correlated residuals, item difficulties, and the fit within in the nomological net. Specifically, items with high standardized loadings were prioritized as particularly indicative of their respective strategies. Loadings were therefore used as the primary criterion for selecting the three items per strategy. For these three items, other criteria were also reviewed, and loading patterns were re-evaluated using three-item measurement models. Additional models considering the wording, similarity, and distribution of items were specified and assessed. In some cases, multiple three-item model suggestions were generated per strategy.

Because fit indices for the just identified three-item measurement models could not be extracted, these models were directly tested within a joint model that included all ER strategies. Following Lee et al. ^9^ and aligning with our goal of separately assessing each strategy, a five-factor model was specified (Model A). In this model, all items loaded onto their respective superordinate factors. Factors were allowed to correlate. For some strategies, multiple three-item models were tested as described above. The different joint models were compared based on their fit indices and factor loadings. The best-fitting-model was selected, with a comparison based on Vignette 1. Modification indices were used to identify potential sources of misfit, particularly in the form of cross-loadings. If an item loaded onto a strategy other than the one it was theoretically assumed to belong to, it was replaced by another item from the same strategy. The resulting model formed the final ERQ State-Short.

D.2 Results

### D.2.1. Measurement Model: Acceptance

The original measurement model containing all six ERQ State items did not fit the data adequately using WLSMV (RMSEA excluded): χ² (9, *N* = 105) = 51.14, *p* < .001; CFI = .877; RMSEA = .212, 90% CI [.158, .270]; SRMR = .100; WRMR = 0.908. Similarly, fit under MLR was unsatisfactory: χ² (9, *N* = 105) = 31.83, *p* < .001; CFI = .802; RMSEA = .155, 90% CI [.100, .215]; SRMR = .088. Allowing for correlated residuals between ERQ14 and ERQ24 improved the fit under WLSMV (RMSEA excluded): χ² (8, *N* = 105) = 27.08, *p* = .001; CFI = .944; RMSEA = .151, 90% CI [.091, .216]; SRMR = .068; WRMR = 0.647. However, the same modification did not yield an acceptable model fit under MLR: χ² (8, *N* = 105) = 17.85, *p* = .022; CFI = .915; RMSEA = .108, 90% CI [.039, .176]; SRMR = .061. After adding correlated residuals between ERQ04 and ERQ09, model fit under MLR became acceptable: χ² (7, *N* = 105) = 10.87, *p* = .144; CFI = .966; RMSEA = .073, 90% CI [.000, .155]; SRMR = .048. Standardized loadings for both WLSMV and MLR are presented in Table A1. Note that the loadings for WLSMV refer to the model containing one modification, while those for MLR refer to the model containing two modifications. The items ERQ09, ERQ24, and ERQ29 were selected for testing the joint model based on their loadings and to minimize redundancy in item content.

### D.2.2. Measurement Model: Avoidance

The original measurement model for avoidance did not show a significant χ² under WLSMV, but the fit indices were acceptable (excluding RMSEA): χ² (9, *N* = 105) = 58.36, *p* < .001; CFI = .961; RMSEA = .230, 90% CI [.176, .287]; SRMR = .067; WRMR = 0.847. No modification was applied. Under MLR, the original model did not fit the data well: χ² (9, *N* = 105) = 28.98, *p* = .001; CFI = .915; RMSEA = .145, 90% CI [.094, .200]; SRMR = .054. Allowing for correlated residuals between ERQ17 and ERQ27 improved model fit under MLR: χ² (8, *N* = 105) = 10.05, *p* = .262; CFI = .991; RMSEA = .049, 90% CI [.000, .124]; SRMR = .031. Standardized loadings for both WLSMV and MLR are reported in Table A1. As all loadings were significant, multiple three-item models were tested within the joint model containing the shortened scales of all strategies.

### D.2.3. Measurement Model: Distraction

The original measurement model containing for the Distraction strategy did not fit well using WLSMV (RMSEA excluded): χ² (9, *N* = 105) = 45.24, *p* < .001; CFI = .816; RMSEA = .197, 90% CI [.142, .256]. SRMR = .113; WRMR = 0.963. Under MLR, the model was similarly inadequate: χ² (9, *N* = 105) = 30.16, *p* < .001; CFI = .721; RMSEA = .150, 90% CI [.095, .208]; SRMR = .089. Allowing for correlated residuals between ERQ05 and ERQ10improved the fit under WLSMV and MLR: WLSMV (RMSEA excluded): χ² (8, *N* = 105) = 23.03, *p* = .003; CFI = .924; RMSEA = .134, 90% CI [.072, .200]; SRMR = .080; WRMR = 0.666; MLR: χ² (8, *N* = 105) = 13.37, *p* = .100; CFI = .929; RMSEA = .080, 90% CI [.000, .151]; SRMR = .068. Standardized loadings in Table A1 refer to the model containing this one modification. For testing in the joint model, ERQ20, ERQ25, and ERQ30 were selected, as other combinations showed negative residual variances, non-significant loadings, or estimation problems.

### D.2.4. Measurement Model: Reappraisal

The original model for Reappraisal did not fit the data well under WLSMV and MLR: WLSMV (RMSEA excluded): χ² (9, *N* = 105) = 41.35, *p* < .001; CFI = .753; RMSEA = .186, 90% CI [.131, .245]; SRMR = .117; WRMR = 0.966;MLR: χ² (9, *N* = 105) = 23.43, *p* = .005; CFI = .752; RMSEA = .124, 90% CI [.066, .183]; SRMR = .086. After allowing for correlated residuals between ERQ01 and ERQ06, the fit under both WLSMV and MLR improved: WLSMV(RMSEA excluded): χ² (8, *N* = 105) = 19.85, *p* = .011; CFI = .909; RMSEA = .119, 90% CI [.054, .187]; SRMR = .079; WRMR = 0.607;MLR: χ² (8, *N* = 105) = 12.74, *p* = .121; CFI = .918; RMSEA = .075, 90% CI [.000, .149]; SRMR = .058. Standardized loadings in Table A1 refer to the model containing the modification mentioned above. For the joint model, ERQ06, ERQ16, and ERQ26 were selected based on their significant loadings and varied item beginnings.

### D.2.5. Measurement Model: Rumination

The original measurement model containing all six ERQ State items produced a significant χ² under WLSMV but exhibited a good fit according to the other indices (RMSEA excluded): χ² (9, *N* = 105) = 18.16, *p* = .033; CFI = .982; RMSEA = .099, 90% CI [.027, .165]; SRMR = .046; WRMR = 0.436. Similarly, under MLR, the model exhibited a good fit: χ² (9, *N* = 105) = 11.71, *p* = .230; CFI = .985; RMSEA = .054, 90% CI [.000, .131]; SRMR = .036. Based on the loadings and difficulties, different three-item models were tested in a joint model that included the shortened scales for all other ER strategies.

### D.2.6. Joint Model of the Shortened Scales

For avoidance and rumination, various three-item measurement models were tested in a joint model that included the shortened scales of all five ER strategies. For acceptance, distraction, and reappraisal, the items mentioned earlier were used. Upon comparing fit indices descriptively using WLSMV, the model containing ERQ07, ERQ12, and ERQ22 for avoidance and ERQ18, ERQ23, and ERQ28 for rumination, was selected. However, it showed some indices that were outside the acceptable fit range using WLSMV (RMSEA excluded): χ² (80, *N* = 105) = 193.91, *p* < .001; CFI = .916; RMSEA = .117, 90% CI [.096, .138]; SRMR = .099; WRMR = 0.989. Under MLR, the indices approached acceptable thresholds: χ² (80, *N* = 105) = 123.18, *p* = .001; CFI = .914; RMSEA = .072, 90% CI [.046, .096]; SRMR = .090. Modification indices indicated that item ERQ24 from the Acceptance scale may be a source of misfit due to cross-loading, prompting its substitution with ERQ19. The fit of the resulting model was close to acceptable under WLSMV and good under MLR (fit indices and loadings for this model are reported in the results section for research question 1). Further inspection of modification indices under WLSMV revealed cross-loadings of ERQ16 and ERQ06 from the Reappraisal scale, with the highest modification index suggesting a cross-loading from ERQ16 onto the distraction factor. Substituting ERQ16 with other reappraisal items, however, led to one of the following issues: non-significant loadings, a non-positive definite covariance matrix for the latent variables, or a model containing items with correlated residuals specified in the measurement model for reappraisal. Therefore, the final ERQ State-Short model retained ERQ16, as this choice provided a reasonable balance between statistical properties and item content.

**Table D1**

*Standardized Loadings for the Extended Pool of ERQ State Items in Their Separate Measurement Models During Vignette 1 (Social Anxiety Condition)*

| ERQ State measurement model | Factor loadings | |
| --- | --- | --- |
|  | WLSMV | MLR |
| Strategy: acceptance ^a^ |  |  |
| ERQ04 Ich kann mich nur schwer damit abfinden, wie es mir gerade geht. (R) | .73^***^ | .57^***^ |
| ERQ09 Ich akzeptiere, wie es mir gerade geht. | –.87^***^ | –.69^***^ |
| ERQ14 Ich denke mir, dass es vorbei gehen wird. | .00 | .06 |
| ERQ19 Ich akzeptiere, dass ich das Gefühl jetzt nicht ändern kann. | –.48^***^ | –.47^***^ |
| ERQ24 Ich denke, dass ich die Situation akzeptieren muss. | –.53^***^ | –.46^***^ |
| ERQ29 Es ist ok, wie ich mich fühle. | –.76^***^ | –.80^***^ |
| Strategy: avoidance ^b^ |  |  |
| ERQ02 Ich würde am liebsten der Situation entfliehen. | .81^***^ | .79^***^ |
| ERQ07 Ich hätte die Situation gerne vermieden. | .86^***^ | .81^***^ |
| ERQ12 Es fällt mir schwer die Situation auszuhalten. | .81^***^ | .78^***^ |
| ERQ17 Ich würde dafür sorgen, nicht in eine solche Situation zu kommen. | .78^***^ | .59^***^ |
| ERQ22 Ich hätte mich gerne zurückgezogen.  ERQ27 Ich würde zukünftig solche Situationen vermeiden. | .87^***^  .82^***^ | .86^***^  .69^***^ |
| Strategy: distraction ^c^ |  |  |
| ERQ05 Ich lenke mich in Gedanken von der Situation hier ab.  ERQ10 Ich stelle mir vor, woanders zu sein.  ERQ15 Ich bin völlig konzentriert auf die Situation hier. (R) | .43^***^  .41^***^  –.0 | .42^**^  .37^**^  .02 |
| ERQ20 Ich denke an angenehme Dinge, die nichts mit der Situation zu tun haben. | .96^***^ | .97^***^ |
| ERQ25 Ich denke an etwas anderes als daran, wie ich mich fühle. | .52^***^ | .41^**^ |
| ERQ30 Ich denke an etwas, das ich gerne tue. | .54^***^ | .48^**^ |
| Strategy: reappraisal ^c^ |  |  |
| ERQ01 Ich versuche, an der Situation etwas Gutes zu finden.  ERQ06 Ich versuche, das Beste aus der Situation zu machen.  ERQ11 Ich versuche, die Situation als Herausforderung zu sehen. | .27^*^  .37^***^  .32^**^ | .25  .40^*^  .28 |
| ERQ16 Ich sage mir, dass das alles doch gar nicht so schlimm ist. | .68^***^ | .64^***^ |
| ERQ21 Ich sage mir, dass ich es beim nächsten Mal besser machen kann. | .45^***^ | .40^**^ |
| ERQ26 Ich sage mir, dass mein Wert als Person davon nicht abhängt. | .59^***^ | .51^**^ |
| Strategy: rumination ^d^ |  |  |
| ERQ03 Ich überlege immer wieder, warum ich mich gerade so fühle, wie ich mich fühle. | .72^***^ | .64^***^ |
| ERQ08 Ich denke immer wieder darüber nach, was jetzt passieren könnte. | .71^***^ | .68^***^ |
| ERQ13 Ich denke nicht besonders über meine Gefühle nach. (R) | –.57^***^ | –.52^***^ |
| ERQ18 Ich habe das Gefühl, keine Kontrolle über meine Gedanken zu haben. | .85^***^ | .80^***^ |
| ERQ23 Ich denke, „Warum reagiere ich immer so?“  ERQ28 Ich denke im Anschluss an die Situation ganz viel darüber nach, was passiert ist. | .83^***^  .70 ^***^ | .78^***^  .66^***^ |

*Note. N* = 105. Items are presented in their original German form as used in the study. Reverse-scored items are indicated with an (R). WLSMV = weighted least squares means and variance adjusted estimator; MLR = robust maximum likelihood estimator.

^a^ Loadings for WLSMV refer to a measurement model with one modification; loadings for MLR refer to a measurement model with two modifications. ^b^ Loadings for WLSMV refer to a measurement model with no modification; loadings for MLR refer to a measurement model with one modification. ^c^ Loadings are based on the measurement model with one modification. ^d^ Loadings are based on the measurement models with no modification.

^*^*p* < .05. ^**^*p* < .01. ^***^*p* < .001.

# **Appendix E - Explorative Inspection of Other Joint Models**

E.1 Other Joint Models Inspected

In addition to Model B, all other models examined were based on prior research (see ^10^ for an overview). The following joint models underwent further exploration:

- Model B: A hierarchical model where, due to their high interfactor correlations, all ER strategies loaded onto a superordinate ER factor.
- Model C ^9,11^: Variations of a two-factor model in which items associated with putatively adaptive and maladaptive strategies were allocated to separate adaptive and maladaptive factor, respectively. Specifically, reappraisal and acceptance were classified as adaptive strategies, while rumination and avoidance were treated as maladaptive strategies. Given the mixed empirical evidence for distraction (e.g., ^10,12^), it was modeled both as adaptive (Model C-a) and maladaptive (Model C-b) in two distinct variations.
- Model D ^10^: A two-factor model in which items from cognitive and behavioral strategies loaded onto a cognitive or behavioral factor, respectively. In this model, avoidance was specified as a behavioral strategy, as it more closely aligns with behavioral avoidance in the current study. Acceptance, reappraisal, and rumination were considered cognitive strategies. Given that distraction in our study aligns more with cognitive distraction as defined by Naragon-Gainey et al. ^10^, it was categorized as a cognitive strategy as well;
- Model E ^10,11^: Variations of a model based on Gross’ Process Model of Emotion Regulation ^13^. Specifically, more avoidance was considered as situation selection, given its closer relationship to behavioral avoidance in our study. Distraction and rumination were modeled as attentional deployment, while reappraisal was specified as cognitive change. Acceptance was modeled either as cognitive change (Model E-a) or response modulation (Model E-b), resulting in two distinct versions of the model ^10^.

It is important to note that while other models have been proposed in previous research (e.g., ^10,11^), they were either not applicable to the present data or were unable to clearly categorize strategies into one factor, and thus not discussed further.

E.2 Results

### E.2.1. Model B: Hierarchical Model

Model B did not produce a satisfactory fit using WLSMV (RMSEA excluded): χ² (85, *N* = 105) = 203.09, *p* < .001; CFI = .912; RMSEA = .116, 90% CI [.095, .136]; SRMR = .109; WRMR = 1.059. It fit significantly worse than the Model A, Δχ² (5, *N* = 105) = 40.01, *p* < .001. Notably, distraction did not load significantly onto the superordinate ER factor, with its loading close to zero. Under MLR, some fit indices indicated an acceptable fit, whereas others did not: χ² (85, *N* = 105) = 120.14, *p* = .007; CFI = .926; RMSEA = .063, 90% CI [.035, .087]; SRMR = .099. Model comparisons again revealed that Model B fitted significantly worse than Model A, Δχ² (5, *N* = 105) = 20.19, *p* = .001. Furthermore, both the avoidance and distraction factors did not significantly load onto their superordinate ER factor, and one reappraisal item, along with all avoidance items, failed to significantly load onto their respective first-order factors.

### E.2.2. Model C: Adaptive-Maladaptive Model

Both versions of Model C did not produce a satisfactory fit under WLSMV:

Model C-a (distraction as adaptive strategy): χ² (89, *N* = 105) = 321.70, *p* < .001; CFI = .826; RMSEA = .159, 90% CI [.140, .177]; SRMR = .130; WRMR = 1.372. Model C-b (distraction as maladaptive strategy): χ² (89, *N* = 105) = 332.26, *p* < .001; CFI = .818; RMSEA = .162, 90% CI [.144, .181]; SRMR = .130; WRMR = 1.386. In comparison to Model A, the fit was significantly worse for both Model C-a, Δχ² (9, *N* = 105) = 50.68, *p* < .001, and Model C-b, Δχ² (9, *N* = 105) = 37.61, *p* < .001. In both versions, the distraction items failed to significantly load onto either the adaptive or maladaptive factors. The fit for Model C under MLR was also unsatisfactory: Model C-a: χ² (89, *N* = 105) = 173.51, *p* < .001; CFI = .822; RMSEA = .095, 90% CI [.074, .115]; SRMR = .120. Model C-b: χ² (89, *N* = 105) = 174.48, *p* < .001; CFI = .820; RMSEA = .096, 90% CI [.075, .116]; SRMR = .119. Both versions of Model C showed a significantly worse fit than Model A: Model C-a, Δχ² (9, *N* = 105) = 71.20, *p* < .001, and Model C-b: Δχ² (9, *N* = 105) = 63.88, *p* < .001. Again, the distraction items and one reappraisal item failed to significantly load onto their respective adaptive or maladaptive factors.

### E.2.3. Model D: Cognitive-Behavioral Model

Model D did not produce a satisfactory fit using WLSMV (RMSEA excluded): χ² (89,
*N* = 105) = 347.77, *p* < .001; CFI = .806; RMSEA = .167, 90% CI [.149, .186]; SRMR = .135; WRMR = 1.435. It also showed significantly worse fit than Model A, Δχ² (9, *N* = 105) = 45.94, *p* < .001. Furthermore, the distraction items did not significantly load onto the cognitive factor. Using MLR, the fit was similarly unsatisfactory: χ² (89, *N* = 105) = 191.72, *p* < .001; CFI = .784; RMSEA = .105, 90% CI [.085, .125]; SRMR = .121. Again, Model D showed a significantly worse fit than Model A, Δχ² (9, *N* = 105) = 80.13, *p* < .001. The distraction items and one reappraisal item did not significantly load onto the cognitive factor.

### E.2.4. Model E: Gross’ Process Model

Both versions of Model E, based on Gross’ Process Model of Emotion Regulation, failed to produce a satisfactory fit under WLSMV: Model E-a (acceptance as cognitive change): χ² (87, *N* = 105) = 325.60, *p* < .001; CFI = .821; RMSEA = .162, 90% CI [.144, .181]; SRMR = .129; WRMR = 1.374. Model E-b (acceptance as response modulation): χ² (84, *N* = 105) = 321.68, *p* < .001; CFI = .822; RMSEA = .165, 90% CI [.146, .184]; SRMR = .127; WRMR = 1.357. In comparison to Model A, both Model E-a and Model E-b showed significantly worse fits: Model E-a: Δχ² (7, *N* = 105) = 46.49, *p* < .001; Model E-b: Δχ² (4, *N* = 105) = 46.54, *p* < .001. In both models, the distraction items did not load onto their respective factors. Under MLR, the fit was also not satisfactory: Model E-a: χ² (87, *N* = 105) = 167.88, *p* < .001; CFI = .830; RMSEA = .094, 90% CI [.073, .115]; SRMR = .119; Model E-b: χ² (84, *N* = 105) = 161.76, *p* < .001; CFI = .836; RMSEA = .094, 90% CI [.072, .115]; SRMR = .117. Both models showed a significantly worse fit than Model A: Model E-a, Δχ² (7, *N* = 105) = 56.03, *p* < .001; Model E-b: Δχ² (4, *N* = 105) = 57.80, *p* < .001. In both models, the distraction items and one reappraisal item did not significantly load onto their respective factors.

# **References**

1. Rönkkö, M. & Cho, E. An Updated Guideline for Assessing Discriminant Validity. *Organ. Res. Methods* **25**, 6–14 (2022).

2. Wu, H. & Estabrook, R. Identification of Confirmatory Factor Analysis Models of Different Levels of Invariance for Ordered Categorical Outcomes. *Psychometrika* **81**, 1014–1045 (2016).

3. Liu, Y. *et al.* Testing measurement invariance in longitudinal data with ordered-categorical measures. *Psychol. Methods* **22**, 486–506 (2017).

4. Gerosa, T. Measurement Invariance with Ordered Categorical Variables: Applications in Longitudinal Survey Research. in *Measurement Error in Longitudinal Data* (eds. Cernat, A. & Sakshaug, J. W.) 259–288 (Oxford University PressOxford, 2021). doi:10.1093/oso/9780198859987.003.0011.

5. Svetina, D., Rutkowski, L. & Rutkowski, D. Multiple-Group Invariance with Categorical Outcomes Using Updated Guidelines: An Illustration Using M *plus* and the lavaan/semTools Packages. *Struct. Equ. Model. Multidiscip. J.* **27**, 111–130 (2020).

6. Cheung, G. W. & Rensvold, R. B. Evaluating Goodness-of-Fit Indexes for Testing Measurement Invariance. *Struct. Equ. Model. Multidiscip. J.* **9**, 233–255 (2002).

7. Bowen, N. K. & Masa, R. D. Conducting Measurement Invariance Tests with Ordinal Data: A Guide for Social Work Researchers. *J. Soc. Soc. Work Res.* **6**, 229–249 (2015).

8. Ziegler, M. Comments on Item Selection Procedures. *Eur. J. Psychol. Assess.* **30**, 1–2 (2014).

9. Lee, D. J., Witte, T. K., Weathers, F. W. & Davis, M. T. Emotion Regulation Strategy Use and Posttraumatic Stress Disorder: Associations Between Multiple Strategies and Specific Symptom Clusters. *J. Psychopathol. Behav. Assess.* **37**, 533–544 (2015).

10. Naragon-Gainey, K., McMahon, T. P. & Chacko, T. P. The structure of common emotion regulation strategies: A meta-analytic examination. *Psychol. Bull.* **143**, 384–427 (2017).

11. Aldao, A., Nolen-Hoeksema, S. & Schweizer, S. Emotion-regulation strategies across psychopathology: A meta-analytic review. *Clin. Psychol. Rev.* **30**, 217–237 (2010).

12. Katz, B. A., Lustig, N., Assis, Y. & Yovel, I. Measuring regulation in the here and now: The development and validation of the State Emotion Regulation Inventory (SERI). *Psychol. Assess.* **29**, 1235–1248 (2017).

13. Gross, J. J. The Extended Process Model of Emotion Regulation: Elaborations, Applications, and Future Directions. *Psychol. Inq.* **26**, 130–137 (2015).

1. Disregarded items in grey. [↑](#footnote-ref-2)
